# GRACE: protocol for a UK, secondary care, multicentre, assessor-blinded randomised controlled trial with a non-inferiority comparison to evaluate graduated compression stockings as an adjunct to extended duration pharmacological thromboprophylaxis for venous thromboembolism prevention

**DOI:** 10.1136/bmjopen-2024-095482

**Published:** 2025-07-06

**Authors:** Rebecca Lawton, Francine Heatley, Andrew D Beggs, Tamara Everington, Zaed Hamady, Beverley J Hunt, Sara Jasionowska, Maria Kyrgiou, Alexander Liddle, Matthew Machin, John Norrie, Tom Pinkney, Jonathan L Rees, Layla Bolton Saghdaoui, Joseph Shalhoub, Sasha Smith, Simon Toh, Nick Watkin, Linda Williams, Alun Davies

**Affiliations:** 1 Section of Vascular Surgery, Department of Surgery and Cancer, Imperial College London, London, UK; 2 Department of Cancer and Genomic Sciences, University of Birmingham, Birmingham, UK; 3 Hampshire Hospitals NHS Foundation Trust, Winchester, UK; 4 University Hospital Southampton NHS Foundation Trust, Southampton, UK; 5 Thrombosis & Haemophilia Centre, Guy's and St Thomas' NHS Foundation Trust, London, UK; 6 Imperial Vascular Unit, Imperial College Healthcare NHS Trust, London, UK; 7 MSk Lab, Surgery and Cancer, Imperial College London, London, UK; 8 School of Medicine, Dentistry and Biomedical Sciences, Queen's University Belfast, Belfast, Ireland; 9 Institute of Applied Health Research, University of Birmingham, Birmingham, UK; 10 Nuffield Department of Orthopaedics, Rheumatology and Musculoskeletal Sciences, University of Oxford, Oxford, UK; 11 Portsmouth Hospitals NHS Trust, Portsmouth, UK; 12 St George’s University of London, London, UK; 13 Edinburgh Clinical Trials Unit, Usher Institute, University of Edinburgh, Edinburgh, UK

**Keywords:** Thromboembolism, SURGERY, HAEMATOLOGY

## Abstract

**Introduction:**

Venous thromboembolism (VTE) occurs when a blood clot forms in a vein. It is comprised of deep vein thrombosis (DVT) and pulmonary embolism and can be potentially life-threatening. Patients undergoing surgery are at increased risk of developing VTE within hospital admission and 90 days after hospital discharge are collectively known as hospital-acquired thrombosis (HAT). Without the use of thromboprophylaxis, the untreated risk of VTE is reported to be as high as 40–60% in those undergoing major orthopaedic procedures and around 15–40% in the general surgical population.

HAT accounts for around 12 000 deaths per year in the UK. For patients undergoing surgery, there is good evidence for the use of thromboprophylaxis to prevent VTE.

Thromboprophylaxis is available in both pharmacological and mechanical forms. While there is a huge body of evidence demonstrating that pharmacological thromboprophylaxis significantly reduces VTE by 30–65%, the benefit of graduated compression stockings (GCS) has been called into question. The GRACE study (Graduated Compression stocking as an adjunct to Extended duration pharmacological thromboprophylaxis for venous thromboembolism prevention) aims to evaluate the adjuvant benefit of GCS in addition to extended duration pharmacological thromboprophylaxis (EDPTP) for elective surgical patients at highest risk of VTE.

**Methods and analysis:**

GRACE is a pragmatic, multicentre randomised trial of adults undergoing surgery who are at high risk of VTE. Participants are randomised into a 1:1 ratio to either EDPTP and compression stockings (control arm) or EDPTP (intervention arm). Following randomisation, participants will undergo surgery and be followed up centrally at 7, 21–35 and 90 days after their procedure. All participants will be offered a bilateral full lower limb duplex scan at 21–35 days post procedure to capture any asymptomatic DVT.

The trial aims to randomise 8608 participants from around 50 National Health Service (NHS) and non-NHS sites in the UK over a 24-month period. The primary endpoint is any imaging-confirmed incidence of VTE within 90 days of surgery.

**Ethics and dissemination:**

On 20 December 2023, GRACE received favourable ethical approval from the Wales Research Ethics Committee 3 Cardiff (23/WA/0350) and the Health Research Authority (IRAS 333539). The results of the study will be disseminated via peer-reviewed publications, presentation at national and international conferences and to study participants via electronic newsletter and social media channels.

**Trial registration number:**

ISRCTN11667770.

**STRENGTHS AND LIMITATIONS OF THIS STUDY**

- Pragmatic multicentre randomised controlled trial.
- Assessor blind design.
- Adherence is patient reported. Although these measures are low cost and low burden, there are well-documented questions around their validity and precision.
- Centralised follow-up to ease the burden on recruiting centres.

## Introduction

 Patients attending hospital to undergo elective surgical procedures are at higher risk of developing venous thromboembolism (VTE), which includes deep vein thrombosis (DVT) in the legs and pulmonary embolism (PE) in the lungs. VTE occurring within hospital admission and 90 days after hospital discharge is known as hospital-acquired thrombosis (HAT). HAT is common and potentially preventable, yet it is responsible for around 12 000 deaths per year in the UK.^1^ As well as the potential to be life-threatening, VTE may also lead to longer-term sequelae, including the development of the post-thrombotic syndrome (PTS) in around 50% of cases.^2^ PTS is characterised by chronic leg pain, oedema and venous skin changes.^3^ Following acute PE, around 2% of patients develop chronic thromboembolic pulmonary hypertension, resulting in chronic pulmonary hypertension and heart failure.^4 5^ Persistent cardiac insufficiency has also been reported in as many as 13.2% of PE cases.^6^ Symptoms of chest pain, dyspnoea, fatigue, light-headedness, syncope and impaired functional or mental status which persist for more than 3 months after diagnosis are collectively classified as post PE syndrome.^7^ Such long-term complications can cause considerable psychosocial impact on patients, leading to impaired quality of life.^6 8^ They are also burdensome to society due to associated healthcare costs and loss of productivity owing to work-related ill health.^9^,^11^

In 2010, it became mandatory for National Health Service (NHS) hospitals in England to assess all admitted adult patients for VTE risk using a national risk assessment tool and to follow National Institute for Health and Care Excellence (NICE) guidelines on thromboprophylaxis. Several strategies to reduce the risk of VTE are used for patients deemed to be at highest risk on this tool, including extended duration pharmacological thromboprophylaxis (EDPTP) prescribed beyond hospital admission and the use of graduated compression stockings (GCS).

There is compelling high-quality evidence to support the use of EDPTP in this context.^12^ NICE guideline NG89 recommends that certain cohorts of surgical patients should also receive mechanical thromboprophylaxis (usually GCS) in addition to the EDPTP.^13^ Despite this recommendation, there is little evidence to support the use of GCS in addition to EDPTP for this patient population.

A Cochrane Review^14^ reported benefit for GCS; however, only small trials were included (18–440 patients), and all except one trial was published prior to 2001.^15^ Two more recent large randomised controlled trials in stroke and orthopaedic patients were not included in this Cochrane review, neither supported the use of GCS.^16 17^ In 2020, the GAPS trial (Graduated compression as an Adjunct to Pharmacoprophylaxis in Surgery) reported that pharmacological thromboprophylaxis alone is non-inferior to pharmacological thromboprophylaxis and GCS in medium and high risk surgical patients.^18^ However, GAPS excluded the study population examined by the GRACE trial, that is, those at the highest risk of VTE who require postdischarge EDPTP.

## Methods and analysis

### Trial design and setting

**Figure 1:**
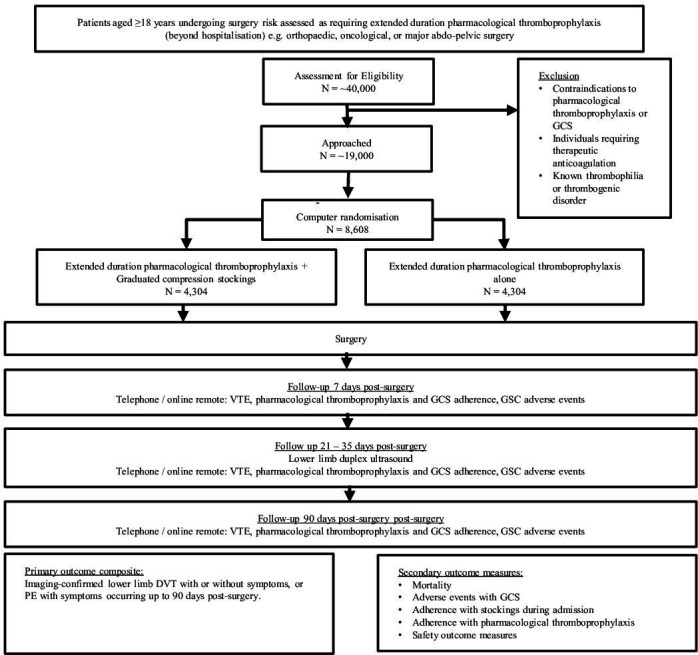
The Consolidated Standards of Reporting Trials flow diagram of the GRACE trial population. DVT, deep vein thrombosis; GCS, graduated compression stockings; GRACE, Graduated Compression stocking as an adjunct to Extended duration pharmacological thromboprophylaxis for venous thromboembolism prevention; VTE, venous thromboembolism.

GRACE is a pragmatic, assessor blind, multicentre randomised trial of adults undergoing surgery who are deemed to be at high risk for VTE. The study has a non-inferiority design, which aims to demonstrate that the intervention arm is not appreciably worse than the control. Randomisation is via a web-based application, stratified by centre and type of surgery. The Consolidated Standards of Reporting Trials flow diagram of the trial population is shown in figure 1. A total of 8608 participants will be recruited from around 36–50 NHS and non-NHS secondary care centres over a 24-month period in the UK. Sites will be selected for their ability to randomise, with a proven track record in conducting research. The development of this trial protocol followed the Standard Protocol Items: Recommendations for Interventional Trials guidelines.^19^

### Recruitment and consent

Potential participants are screened from elective surgical lists or preassessment clinics at recruiting centres. They will be provided with an information sheet and time to discuss any questions or concerns prior to giving consent (online supplemental appendix 1). Once consent is obtained, eligibility is confirmed, and patients can be randomised into the trial. Full details of consenting procedures can be found in the GRACE protocol, and the eligibility criteria are shown in table 1. Screened participants who do not meet the eligibility criteria or decline to participate will be logged pseudonymously along with a minimum data set of age, sex and reason for exclusion. These logs will be reported to the Trial Coordinating Centre for the purposes of monitoring recruitment.

**Table 1:** Eligibility criteria

Inclusion criteria	Exclusion criteria
Adults (≥18 years)	Contraindications to EDPTP or GCS
Participants undergoing elective surgery; risk assessed as requiring EDPTP*	Individuals requiring therapeutic anticoagulation†, for example, anticoagulation for previous DVT
	Known thrombophilia or thrombogenic disorder

* Participants are deemed to require extended duration thromboprophylaxis measures as per local policy in line with National Institute for Health and Care Excellence (NG89) guidelines.

† Antiplatelet therapy, for example, aspirin is not an exclusion.

DVT, deep vein thrombosis; EDPTP, extended duration pharmacological thromboprophylaxis; GCS, graduated compression stockings.

### Randomisation

Following eligibility checks and informed consent procedures, local research staff will randomise participants into a 1:1 ratio to either extended duration thromboprophylaxis alone (intervention arm) or extended duration thromboprophylaxis plus GCS (control arm). Randomisation is via the Research Electronic Data Capture (REDCap) database^20 21^ hosted at the Edinburgh Clinical Trials Unit (UK Clinical Research Collaboration registration number 15).

### Interventions

EDPTP is defined by the protocol as at least 5 days of anticoagulation given post discharge. As this is a pragmatic trial, the length of the prescription and type of anticoagulant will follow usual local practice based on balancing bleeding risk against the risk of developing VTE. Those randomised to receive GCS in addition to the EDPTP will follow local practice in terms of GCS application and duration post surgery. Depending on the type and complexity of surgery and local hospital policy, the duration for the prescription of GCS may be until ambulant, until discharge or longer. This will be fully reported in the results publication.

### Blinding

Due to the nature of the intervention, it is impossible to blind patients or research staff. However, vascular scientists/technologists conducting the 21–35 day lower limb duplex scan are blinded to the treatment allocation. If patients are scanned on clinical suspicion of DVT prior to the study scan, they will be asked to remove GCS if they are still wearing them to prevent unblinding. If this scan is positive for DVT, this will equate to a positive event, and a research scan is not required.

### Primary endpoint

The primary outcome is a binary combined endpoint of imaging-confirmed lower limb DVT with or without symptoms, or imaging-confirmed symptomatic PE, occurring up to 90 days post surgery.

### Secondary endpoints

Secondary outcomes, defined as occurring within 90 days of surgery, include:

- Mortality.
- Adverse events (AEs) related to GCS.
- Adherence with GCS.*
- Adherence with EDPTP.*
- Safety outcome measures, including major bleeding.

*Adherence with GCS will be assessed in comparison to the advice provided by the local clinical team at the time of discharge, that is, if advised to wear GCS every day for the full duration of extended thromboprophylaxis, then this timepoint will be the level of full compliance for comparison. However, if participants are advised to wear GCS, while an inpatient only, then this will be used as the level of full compliance. Adherence with EDPTP is self-reported. Participants will be asked at each follow-up if they are still taking their EDPTP and if they are taking it/took it as directed by their local clinical team.

### Sample size

The null hypothesis for non-inferiority designs, is that the randomised groups differ. VTE rates in the absence of treatment (ie, no GCS or pharmacological thromboprophylaxis) are reported to be as high as 40% for joint replacement and 13% for abdominal and pelvic surgery.^22 23^ Based on NHS England Hospital Episode Statistics data^24^ and a summation analysis, we have assumed an 8% VTE rate for standard of care. GRACE is designed to have 90% power at a one-sided 2.5% level of significance, which means the study requires an unadjusted sample size of 7736 to detect a non-inferiority margin of 2%, assuming the standard of care (EDPTP and GCS) VTE rate at 90 days is 8%. The study will follow a group sequential design with two interim analyses at specific recruitment milestones (at 40% and 65% and a final analysis at 100% if the trial is not stopped early). This will provide flexibility to stop early for overwhelming evidence of effectiveness or for futility. An asymmetric two-sided non-binding Hwang-Shih-DeCani spending function inflates the sample size to 8264. From the GAPS trial, we know 4% of participants had a missing primary outcome,^18^ so we have adjusted the maximum expected sample size up to 8608.

### Internal pilot of feasibility and progression criteria

An internal pilot of feasibility has been planned after 9 months of recruitment to ensure the trial is on track to reach recruitment milestones. At this point, if trial recruitment is under target, there is the option to set up an extra 10 sites. The trial steering committee (TSC) may consider stopping if fewer than 18 sites are on board and may consider adapting the trial if between 18 and 35 sites are on board. A detailed description of recruitment scenarios can be found in the protocol.

### Follow-up

Participants will be followed up centrally at 7 days, between 21 and 35 days, and 90 days post surgery via email or telephone. Between 21 and 35 days post intervention, participants are invited by their local recruiting hospital to undergo a bilateral full lower-limb duplex scan of their legs to identify any asymptomatic DVT. Imaging-confirmed symptomatic DVT can be reported at any time during the study period. A positive finding of DVT or PE on the scan will be treated and followed up in line with local hospital policy.

### Adverse events (AEs)

Only AEs related to the study intervention (GCS) will be recorded at each follow-up. REDCap has built-in triggers that will alert the coordinating centre to any patient-reported AEs or serious AEs (SAEs). Participants who report a treatment-related AE that requires further investigation or follow-up will be advised to consult with their GP/relevant clinical team. All SAEs will be reported to the independent data monitoring committee (iDMC). The sponsor (Imperial College London) holds relevant clinical trials insurance.

### Change of status

The investigators understand that the nature and extent of patient participation may change during the study. Local research sites are encouraged to discuss any changes to participation using the Persevere Principles,^25^ as participants may be willing to contribute to the study in a reduced or altered manner.

### Monitoring

An iDMC and a TSC have been established in line with the National Institute for Health Research (NIHR) Health Technology Assessment (HTA) terms of reference. These committees will oversee trial conduct and monitor study data and safety.

At the point of entry into the study, patient details will be pseudoanonymised via a study ID and stored on REDCap. A local key held by site principal investigators will link this ID to clinical patient records. Data will be filed for 10 years as per local policy and then deleted.

### Data analysis

All statistical analyses of primary and secondary endpoints will be governed by a statistical analysis plan written by the study statistician and approved by the TSC and iDMC prior to viewing any unblinded data. The main analysis will follow an intention-to-treat principle, comparing rates of VTE occurring any time up to 90 days. Due to the non-inferiority design, a suitably specified per protocol analysis will be conducted.

Secondary outcome analysis will be conducted in a similar manner, and safety data will be summarised descriptively.

### Ethics and dissemination

GRACE received favourable ethical approval from the Wales Research Ethics Committee 3 Cardiff (23/WA/0350) and the Health Research Authority (IRAS 333539). The study has been adopted onto the UK Clinical Research Network (UKCRN) portfolio (CPMS 60092). The intervention arm involves the study of a medicinal product, but Medicines and Healthcare products Regulatory Agency (MHRA) approval is not required as the product will be used within its licensed indication. Results will be disseminated in a peer-reviewed journal and presented at national and international conferences and to study participants via electronic newsletter and social media channels.

### Patient and public involvement (PPI)

PPI commenced at the study development stage. Two expert PPI coapplicants supported the grant application process, offering feedback on the suitability of the study design, the research question, a plain English summary and patient-facing documents. We also engaged with the wider VTE community, gaining their opinions on the study design via a short online survey. Based on the findings of the survey, we made a few alterations to the grant application.

In line with NIHR PPI resources, the NIHR Collaboration for Leadership in Applied Health Research Toolkit, we will continue our PPI work throughout the study, particularly if there are difficulties recruiting or retaining participants. Our expert PPI coapplicants will be invited to attend TSC meetings, and PPI will also play an important part in the final study write-up and dissemination.

### Data sharing

The datasets generated and/or analysed during this study will be included in the subsequent results publication and available for access on a case-by-case basis following a written request to the chief investigator.

## Supplementary material

10.1136/bmjopen-2024-095482 online supplemental file 1

10.1136/bmjopen-2024-095482 online supplemental file 2
