# Supplementary material for: GRACE: protocol for a UK, secondary care, multicentre, assessor-blinded randomised controlled trial with a non-inferiority comparison to evaluate graduated compression stockings as an adjunct to extended duration pharmacological thromboprophylaxis for venous thromboembolism prevention
Source: BMJ Open. 2025 Jul 6;15(7):e095482. doi: 10.1136/bmjopen-2024-095482 (PMC12230952; doi:10.1136/bmjopen-2024-095482)
Supplement: online supplemental file 1 [file bmjopen-15-7-s001.pdf]

GRACE

Graduated Compression stocking as an adjunct to Extended duration pharmacological thromboprophylaxis for venous thromboembolism prevention

(The GRACE multi-centre randomised controlled trial)

V2.0, 15/05/2024

SPONSOR: Imperial College London
FUNDER: National Institute for Health Research (NIHR) – Health Technology Assessment (HTA)
STUDY COORDINATION CENTRE: Imperial College London

Revision History

Protocol Version	Date	Amendments
V 2.0		Change of Senior statistician to Dr Linda Williams (Pg 2/3)<br>Update to allow recruitment from non-NHS sites (Pg 21, 31) and 'An additional sensitivity analysis of NHS-only patients (including patients referred by their GPs to non-NHS sites) will be conducted to ensure generalisability to a NHS population.', Pg 31)<br>Clarify that full lower limb ultrasound is bilateral and includes groin to ankle (Pg 26/27)<br>Clarify that both above and below knee stockings are allowed and duration of use is as per local policy (Pg 24)<br>Clarify that confirmation of VTE by imaging is required if patient self reports a VTE (Pg 27)<br>Clarify that 'Recruitment will continue while the interim data is analysed.' (Pg 31)<br>Addition of section to detail the collection of protocol deviations and violations and serious breaches (Pg 38)<br>The correction of typological errors (≥18 years, Pg 20), EDTPT post discharge, Pg 24), removal of 'witnessed' for verbal consent, Pg 25), ('since last contact Pg 27), Addition of 'randomisation' Pg 38)
V 1.0	30/11/2023	Protocol approved by the REC

Protocol authorised by:

**Name & Role**	**Date**	**Signature**
Professor Alun Davies<br>Chief investigator	01/07/2024	[signature]
Dr Linda Williams<br>Lead Statistician	01/07/2024	Linda Williams

Study Management Group

Chief Investigator:

Professor Alun Davies
Imperial College London, Academic Section of Vascular Surgery
Room 3, 4th Floor East Wing, Charing Cross Hospital
Fulham Palace Road, London, W6 8RF
Email: a.h.davies@imperial.ac.uk

Senior Statistician:

Dr Linda Williams
Senior Statistician
Edinburgh Clinical Trials Unit
Usher Institute- University of Edinburgh
5-7 Little France Road, Nine Edinburgh BioQuarter
Edinburgh, EH16 4UX
Email: Linda.Williams@ed.ac.uk

Co-investigators:

Mr Joseph Shalhoub	Honorary Clinical Senior Lecturer	Imperial College London
Professor John Norrie	Director of Edinburgh Clinical Trials Unit	University of Edinburgh
Mr Matthew Machin	Academic Clinical Research Fellow	Imperial College London
Professor Beverley Hunt	Professor of Thrombosis and Haemostasis	Guy's and St Thomas' NHS Foundation Trust

Dr Tamara Everington	Consultant in Haemostasis and Thrombosis	Hampshire Hospitals NHS Foundation Trust
Professor Jonathan Rees	Professor of Orthopaedic Surgery and Musculoskeletal Science	University of Oxford
Dr Sara Jasionowska	NIHR Academic Foundation Programme Doctor	Imperial College London
Professor Andrew Beggs	Professor of Surgery and Cancer Genetics	University of Birmingham
Professor Maria Kyrgiou	Professor of Gynaecological Oncology	Imperial College London
Mr Alexander Liddle	Clinical Senior Lecturer in Orthopaedics / Consultant Orthopaedic Surgeon	Imperial College London
Professor Thomas Pinkney	Professor of Surgical Trials and Honorary Consultant General and Colorectal Surgeon	University of Birmingham
Professor Simon Toh	Professor of Surgery & Consultant Surgeon	Portsmouth Hospitals University NHS Trust
Professor Nick Watkin	Professor of Urology	St George's, University of London
Mr Zaed Hamady	Consultant General Surgeon	University Hospital Southampton NHS Foundation Trust
Ms Rebecca Lawton	Clinical Trial Manager	Imperial College London
Miss Sasha Smith	Clinical Trial Manager	Imperial College London
Ms Layla Bolton	Clinical Nurse Specialist	Imperial College London
Ms Annya Stephens-Boal	PPI representative	
Mr Andy Steptow	PPI representative	

Study Coordination Centre

For general queries, supply of study documentation, and collection of data, please contact:

Study Coordinator: Dr Francine Heatley
Imperial College London
Room 3E, 4th Floor East Wing
Charing Cross Hospital
Fulham Palace Road
London W6 8RF
Tel: +44 (0)203 311 7371
E-mail: gracetrial@imperial.ac.uk

Clinical Queries

Clinical queries should be directed to the Trial Manager at gracetrial@imperial.ac.uk who will direct the query to the appropriate person

Sponsor

Imperial College London is the main research Sponsor for this study. For further information regarding the sponsorship conditions, please contact the Head of Regulatory Compliance at:

Research Governance and Integrity Team
Imperial College London and Imperial College Healthcare NHS Trust
Room 215, Level 2, Medical School Building
Norfolk Place
London, W2 1PG
Tel: **0207 594 1862**
Imperial College - Research Governance and Integrity Team (RGIT) Website

Funder

This study is funded by the National Institute for Health Research (NIHR) Health Technology Assessment programme (NIHR155294). The views expressed are those of the author(s) and not necessarily those of the NIHR or the Department of Health and Social Care.

This protocol describes the GRACE study and provides information about procedures for entering participants. Every care was taken in its drafting, but corrections or amendments may be necessary. These will be circulated to investigators in the study. Problems relating to this study should be referred, in the first instance, to the Chief Investigator.

This study will adhere to the principles outlined in the UK Policy Frame Work for Health and Social Care Research. It will be conducted in compliance with the protocol, the Data Protection Act and other regulatory requirements as appropriate.

KEYWORDS

Venous Thromboembolism, graduated compression stockings, deep venous thrombosis, pulmonary embolism

Table of Contents

Table of Contents	Page No
1. INTRODUCTION	- 10 -
1.1. BACKGROUND	- 10 -
1.2. RATIONALE FOR CURRENT STUDY	- 11 -
2. STUDY OBJECTIVES	- 13 -
2.1 PRIMARY OUTCOME	- 14 -
2.2 SECONDARY OUTCOMES	- 14 -
3. STUDY DESIGN	- 14 -
4. PARTICIPANT ENTRY	- 20 -
4.1. INCLUSION CRITERIA	- 20 -
4.2. EXCLUSION CRITERIA	- 20 -
4.3. WITHDRAWAL CRITERIA	- 20 -
5. ASSESSMENT AND FOLLOW-UP	- 21 -
5.1 ELIGIBILITY AND ASSESSMENT	- 21 -
5.2 INFORMED CONSENT	- 24 -
5.3 FOLLOW-UP	- 26 -
5.4 END OF STUDY	- 28 -
6. STATISTICS AND DATA ANALYSIS	- 29 -
6.1 Sample size calculation	- 29 -
6.2 Statistical analyses	- 30 -
6.3 Subgroup analyses	- 31 -
6.4 Interim analysis	- 31 -
7. ADVERSE EVENTS	- 32 -
7.1. DEFINITIONS	- 32 -
7.2. REPORTING PROCEDURES	- 33 -
8. REGULATORY ISSUES	- 35 -
8.1. ETHICS APPROVAL	- 35 -
8.2. CONSENT	- 35 -
8.3. CONFIDENTIALITY	- 35 -
8.4. INDEMNITY	- 36 -
8.5. SPONSOR	- 36 -
8.6. FUNDING	- 36 -
8.7. AUDITS	- 37 -
9. DATA MANAGEMENT	- 37 -
10. STUDY MANAGEMENT	- 39 -
11. PUBLICATION POLICY	- 39 -
12. REFERENCES	- 41 -

GLOSSARY OF ABBREVIATIONS

CI	Confidence Interval
CTU	Clinical trials unit
DHRA tool	Department of health risk assessment tool
DOAC	Directly acting Oral AntiCoagulants
DVT	Deep venous thrombosis
EDPTP	Extended duration pharmacological thromboprophylaxis
GAPS	Graduated compression stockings as adjuvant to pharmaco-thromboprophylaxis in elective surgical patients' study
GCS	Graduated compression stocking
HAT	Hospital-acquired thrombosis
ICC	Intraclass correlation
iDMC	Independent data monitoring committee
LMWH	Low-molecular weight heparin
NHS	National Health Service
NICE	National Institute for Health and Care Excellence
PE	Pulmonary embolism
PPI	Patient and public involvement
PTS	Post-thrombotic syndrome
RCT	Randomised Controlled Trial
RGIT	Research Governance and Integrity Team
TEDS	Thromboembolic deterrent stockings
SAP	Statistical Analysis Plan
SMS	Short Messaging Service
SOP	Standard operating procedure
TSC	Trial Steering Committee
VTE	Venous thromboembolism
WHO	World Health Organisation

STUDY SUMMARY

**TITLE**	Graduated Compression stocking as an adjunct to Extended duration pharmacological thromboprophylaxis for venous thromboembolism prevention – the GRACE multi-centre randomised controlled trial
**TRIAL DESIGN**	Assessor-blinded randomised controlled trial with a non-inferiority comparison
**AIMS**	To evaluate the potential benefit of Graduated Compression Stockings (GCS) in addition to extended duration pharmacological thromboprophylaxis (EDPTP) for elective surgical patients at highest risk of venous thromboembolism (VTE)
**OUTCOME MEASURES**	Primary Outcome:<br>Imaging-confirmed lower limb DVT with or without symptoms, or PE with symptoms, occurring up to 90 days post-surgery.<br><br>Secondary outcomes up to 90 days:<br>• Mortality<br>• Adverse events with GCS<br>• Adherence with GCS<br>• Adherence with EDPTP<br>• Safety outcome measures
**POPULATION**	Adults undergoing surgical procedures requiring extended duration thromboprophylaxis measures as described by NICE.
**SAMPLE SIZE**	8,608 participants from approximately 50 sites.
**PATIENT ELIGIBILITY**	Inclusion criteria<br>• Adults (≥18 years of age)<br>• Participants undergoing elective surgery; risk assessed as requiring EDPTP<br><br>Exclusion criteria<br>• Contraindications to EDPTP or GCS*<br>• Individuals requiring therapeutic anticoagulation e.g., anticoagulation for previous DVT<br>• Known thrombophilia or thrombogenic disorder<br><br>***antiplatelet therapy e.g. aspirin is not an exclusion***
**DURATION**	39 months (6 months set-up; 24 months recruitment; 3 months follow-up; 6 months close-down)

1. INTRODUCTION

1.1. BACKGROUND

Hospital-acquired thrombosis (HAT) is defined as any venous thromboembolism (VTE) during admission and within 90 days of hospital discharge (1), and is a term that encompasses both deep vein thrombosis (DVT) and pulmonary embolism (PE). It is estimated that as many as 60% of all VTE cases are hospital related (2). HAT represents a significant cause of preventable mortality, with around 60.4 deaths from VTE per 100,000 hospital admissions reported in the year 2020-2021 within the NHS (1) – equivalent to around 12,300 people dying each year from hospital-caused VTE, which is 10-fold higher than the number dying from road traffic accidents. The UK annual VTE-related mortality prior to implementation of a national programme of thromboprophylaxis, was estimated to be up to 32,000 fatalities, with associated costs as high as £640 million per annum (3).

VTE is a significant cause of long-term disability with subsequent societal and economic consequences (4). Morbidity following a DVT is substantial, with as many as 50% of patients developing post thrombotic syndrome (PTS) (5), characterised by troubling chronic leg pain, oedema, and venous skin changes (6). Crucially, the severity of chronic venous disease is much worse in those with PTS in comparison to primary venous disease and the rate of venous ulceration is as high as 29% (7). Those with PTS have a significantly worse quality of life, which is comparable to those with cancer and congestive heart failure (8, 9). Furthermore, PE is associated in some with life-long functional, physiological and psychological harm (10, 11). Ultimately, PE can cause death during the index event and in an estimated 2% can be complicated by chronic thromboembolic pulmonary hypertension, resulting in chronic pulmonary hypertension and heart failure (12, 13). Persistent cardiac insufficiency have been reported in as many as 13.2% of PE cases (11).

Surgery is a significant risk factor for VTE, with those undergoing lower extremity orthopaedic operations, major oncological surgery or pelvic surgery being at the highest risk (14, 15). Previous studies report the risk of untreated high-risk surgical

patients developing HAT is as high as 40-60% in elective joint replacement and 15-40% in general surgery patients (14, 16). For these patients at highest risk of VTE, the prevention strategies include extended pharmacological thromboprophylaxis (EDPTP) prescribed beyond the hospital stay and provision of graduated compression stockings (GCS). National Institute for Health and Care Excellence (NICE) [NG89] guidelines recommends certain cohorts of surgical patients should receive mechanical thromboprophylaxis, most commonly in the form of GCS, in addition to the EDPTP (17). This cohort encompasses patients undergoing orthopaedic surgery with ongoing lower limb immobilization including elective hip replacement surgery (EDPTP for 28-38 days post-surgery) and elective knee replacement surgery (EDPTP for 14 days post-surgery) in addition to those undergoing major cancer surgery in the abdomen or pelvis (EDPTP for 28 days post-surgery) (17).

However, it is currently unclear if GCS provide additional benefit to EDPTP in this cohort. Recent studies have called this practice into question, the NIHR-funded Graduated compression as an Adjunct to Pharmacoprophylaxis in Surgery (GAPS) trial demonstrated that inpatient pharmacological thromboprophylaxis was non-inferior to inpatient pharmacological thromboprophylaxis in addition to GCS for surgical inpatients deemed to be at medium-high risk of VTE (18). For those requiring EDPTP, there is insufficient evidence to support the provision of GCS. There is uncertainty for healthcare professionals and current practice is potentially leading to avoidable complications and significantly increased NHS costs.

1.2. RATIONALE FOR CURRENT STUDY

There is currently no high-quality evidence to support the use of GCS in addition to EDPTP in patients deemed to be at the highest risk of VTE. A Cochrane Review (19) which reported benefit for GCS included small trials (18-440 patients), only one of which was published this millennium (20), and many of these trials were funded by stocking manufacturers.

Additionally, two large recent trials were excluded from this review in stroke and orthopaedic patients - neither study supported the use of GCS (21, 22). Furthermore,

as noted above, the GAPS trial reported that pharmacological thromboprophylaxis alone is non-inferior to pharmacological thromboprophylaxis and GCS (18). This proposal includes individuals requiring EDPTP, at highest risk, who were excluded from GAPS, hence the results cannot be directly extrapolated (18).

In preparation for this bid, a systematic review and meta-analysis of VTE rates in surgical patients treated with pharmacological thromboprophylaxis in addition to GCS in comparison to pharmacological thromboprophylaxis alone was undertaken in accordance with PRISMA guidelines (23) and following a registered protocol (CRD42017062655), accepted for publication in *Annals of Surgery*). Online searches of the MEDLINE and EMBASE databases were performed returning randomised trials and trial arms reporting the rate of VTE after any surgical procedure, utilising pharmacological thromboprophylaxis alone or pharmacological thromboprophylaxis in addition to GCS. Individual rates of deep venous thrombosis (DVT), pulmonary embolism (PE), and VTE-related mortality were pooled via fixed and random effects models. Clinicaltrials.gov, European Union Clinical Trials, International Standard Randomised Controlled Trial Number Registry were also searched for active trials in this area. This review concluded “no significant differences in rates of DVT, PE and VTE-related mortality between pharmacological prophylaxis alone versus pharmacological prophylaxis with additional GCS”. Subgroup analysis of surgical patients treated with EDPTP pooling data from 27 trial arms was undertaken. When pooling 24 study arms reporting EDPTP alone, including 10,187 participants, the rate of DVT was 10.97%. Analysis of 3 study arms reporting EDPTP in addition to GCS, pooling 651 participants, demonstrated a DVT rate of 7.37%. A single RCT directly comparing EDPTP alone vs EDPTP in addition to GCS reported rates of VTE of 4.8% and 5.5% respectively (OR 0.88, 95% CI: 0.46-1.65, p= 0.69). However, this study reported symptomatic VTE only, and is consequently under powered, hence the lower reported numbers in comparison to screen detected DVT in the aforementioned pooled trial arms. Overall, the additional benefit of GCS is not clear based on existing data.

Highlighting the importance of VTE, NICE alongside the Commissioning for Quality and Innovation (CQUIN), have prioritised VTE reduction strategies over the last decade (24). National initiatives have made VTE risk assessment, such as the Department of Health risk assessment tool (DoH RAT), and subsequent prescription of thromboprophylaxis, routine practice resulting in 96% VTE risk assessment compliance for adult inpatients by 2013 (17, 25). Hence, VTE risk assessment is now an integral part of routine clinical practice, however, there is a lack of high-quality evidence to guide subsequent thromboprophylaxis strategies employed based on these mandated risk assessments. Our detailed feasibility survey revealed that there are currently two established practices within the NHS: those that prescribe additional EDPTP with additional GCS and those that prescribe EDPTP alone.

We undertook a review of the Hospital Episode Statistics data and ascertained that ~270,000 procedures requiring EDPTP are undertaken each year within the NHS (26). The cost of purchasing and applying GCS equates to a minimum of £31.05 per patient episode (18, 27). Based on our evaluation, the costs of GCS on conservative figures for those requiring EDPTP is a minimum of £8.3 million per year. In addition to the financial cost, GCS are burdensome for patients, with complication rates of up to 5%, including discomfort, ulceration, rashes, formation of a constrictive band, ischaemia and abrasions (21, 28). GCS may also pose a falls hazard given their synthetic material construction (29).

With increasing efficiency savings required, we need clear evidence to justify the huge financial resource allocated to GCS. Furthermore, if GCS were to offer benefit in VTE prevention over and above that afforded by EDPTP, this would need to be weighed against their risks and disadvantages.

2. STUDY OBJECTIVES

To evaluate the VTE rates with or without the use of Graduated Compression Stockings (GCS) in addition to giving blood thinning medication post discharge from hospital for surgical patients at highest risk of venous thromboembolism (VTE).

The secondary aims are to determine any difference in adverse events and mortality with or without GCS use.

2.1 PRIMARY OUTCOME

The primary outcome is imaging-confirmed lower limb DVT with or without symptoms, or PE with symptoms, occurring up to 90 days post-surgery.

2.2 SECONDARY OUTCOMES

Secondary outcomes will be defined as occurring within 90 days of surgery.

- Mortality
- Adverse events related to GCS
- Adherence with GCS*
- Adherence with EDPTP
- Safety outcome measures

*Adherence will be assessed in comparison to the advice provided by the local clinical team at the time of discharge i.e., if advised to wear GCS every day for the full duration of extended thromboprophylaxis, then this timepoint will be the level of full compliance for comparison. However, if participants are advised to wear GCS whilst an inpatient only, then this will be used as the level of full compliance.

3. STUDY DESIGN

3.1 TYPE OF STUDY

This is a multi-centre assessor-blinded randomised controlled trial (RCT) consisting of participants undergoing surgical intervention that are deemed to require extended duration thromboprophylaxis with a non-inferiority comparison. Clinicians and participants will be aware of their treatment allocation. Assessors, being those who

perform the lower limb DVT scan and those responsible for collecting follow-up data, will be blinded to the treatment allocation.

Individual participants will be randomised in a 1:1 fashion to one of two thromboprophylaxis strategies:

- EDPTP* in addition to GCS, or
- EDPTP alone

*EDPTP includes any anti-thrombotic agent prescribed at a prophylactic dose for prevention of VTE, including low-molecular weight heparin (LMWH), Directly acting Oral AntiCoagulants (DOACs), or antiplatelet therapy.

Randomisation will be electronic with stratification incorporating centre and type of surgery.

The duration of the study will be 39 months including 6-months set up, 24 months of recruitment, 3 months follow-up and 6 months for analysis and dissemination.

The flow diagram summarising the study design can be seen in **Figure 1**.

Figure 1: Study flow diagram

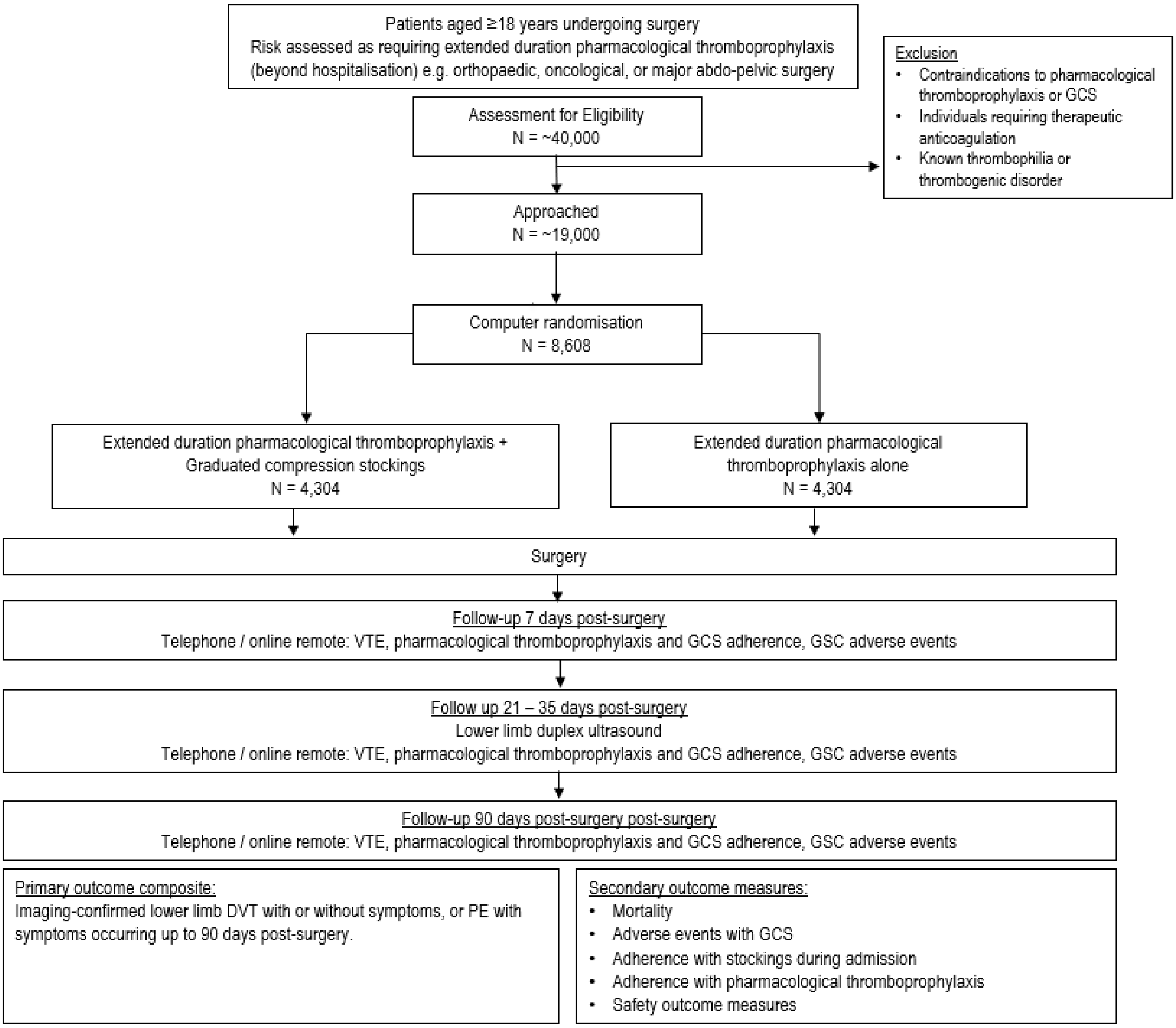

3.2 BLINDING

Assessors, being those who perform the lower limb DVT scan and those responsible for collecting follow-up data, will be blinded to the treatment allocation. If patients in the GCS arm are still wearing stockings at the time of the scan, they will be instructed to remove them prior to entering the scan room and not inform the scanner of their treatment allocation.

3.3 FOLLOW UP

Participants will be followed-up until 90-days post operation.

3.4 INTERNAL PILOT

There will be an internal pilot of feasibility after 9 months of recruitment, (beginning of funding month 7 to end of funding month 15)

In preparation for this application, a search of NHS England HES treatment episodes was undertaken, supplemented by searches of the National Cancer Registration and Analysis Service (26, 39). Treatment episode codes are available upon request. These figures were corroborated with available reports from the Colorectal Cancer Screening Programme. A total of about 270,000 procedures requiring EDPTP are undertaken each year within the NHS. This includes: ~121,410 hip replacements, 87,497 knee replacements, and 55,220 major abdominal / pelvic operations. This equates to 675,000 procedures undertaken across the active recruitment period, with our sample representing as few as 1.3% of cases performed across the UK.

From the feasibility analysis from sites, they stated that they would treat between 30–1000 overall potential participants requiring EDPTP each month. Based on a staggered set-up of approximately 50 sites over 13 months of the recruitment window, at a rate of 4 sites per month to reflect pragmatic trial set up, including 11 months of all sites recruiting at steady state and subsequent 90 days follow-up of the last participant, a total of 890 centre-months recruitment would be accumulated.

Recruiting at a rate of ~9.7 participants per active month per site would reach the sample size by the end of 24-month recruitment window.

By the end of the 9-month internal pilot we will have set up 36 sites and accumulated 166 centre months (assuming on average a site contributes half a month in the month they are set up). As detailed earlier, the 100% recruitment rate is 10 participants per site per month (which accounts for the staggered site set-up). This equates to a target of 1571 participants at the end of the 9-month internal pilot. Although 1571 is equivalent to 18.2% of the overall 8,608 participants, it is indicative that the trial is on track to meet 100% recruitment by the end of 24-month recruitment window (**Table 1**).

If trial recruitment was under target, there is the option to set up an extra 10 sites. A second staggered set-up from month 18 (allowing time from TSC meeting to then achieving confirmation of capacity and capability from local research and development units) of the additional 10 sites has been modelled amounting to 126 additional centre-months by the end of the 24-month window. We would consider stopping if we had less than 18 sites and consider adapting if we had between 18 and 35 sites.

In line with the NIHR-funded GAPS trial(18), we have set reasonable adherence (percentage of actual days / maximum number days, according to local practice) as 80% throughout the index hospital admission and the 7-day adherence at 70%, which has been included in the stop-go criteria. The TSC will meet every 6 months and at the end of month 15 (after 9 months of recruitment allowing 1 month processing time).

Table 1: Internal pilot feasibility assessment at 9 months

**Progression criteria**	**Red**	**Amber**	**Green**
% Threshold	<70	70 – 99.9%	≥100
Trial recruitment (of eligible participants)	<17.9%	17.9 – 25.4%	≥25.5%
Recruitment rate / site / month	<7	7 – 9	≥10
Number of sites opened	<25	25 - 35	≥36
Total number of participants recruited	<1100	1100 – 1570	≥1571
Adherence	<80% inpatient <70% at 7-day follow-up		

4. PARTICIPANT ENTRY

4.1. INCLUSION CRITERIA

- Adults (≥18 years)
- Participants undergoing elective surgery; risk assessed as requiring EDPTP**

** Participants are deemed to require extended duration thromboprophylaxis measures as per local policy in line with NICE [NG89] guidelines.

Examples of procedures from which patients are at highest-risk of VTE include (but are not limited to) (30, 31): orthopaedic surgery (total hip replacement, total knee replacement, colorectal surgery (colectomy, splenectomy), upper gastrointestinal surgery (oesophagectomy, gastrectomy), urological surgery (cystectomy, nephrectomy), and gynaecological oncology surgery (radical hysterectomy, radical trachelectomy).

4.2. EXCLUSION CRITERIA

- Contraindications to EDPTP or GCS
- Individuals requiring therapeutic anticoagulation* e.g., anticoagulation for previous DVT
- Known thrombophilia or thrombogenic disorder

antiplatelet therapy e.g. aspirin is not an exclusion

4.3. WITHDRAWAL CRITERIA

Participants may discontinue study intervention for the following reasons:

- At the request of the participant.
- Adverse event/ Serious Adverse Event
- If the investigator considers that a participant's health will be compromised due to adverse events or concomitant illness that develop after entering the study.

The reason for participant withdrawal should be documented in the Study Completion CRF in REDCap. Participants that are withdrawn or lost to follow-up do not need to be replaced.

5. ASSESSMENT AND FOLLOW-UP

5.1 ELIGIBILITY AND ASSESSMENT

This trial will take place in approximately 36 to 50 NHS and non NHS sites offering day case and short stay surgical services. Patients from a variety of surgical specialties will be included in this pragmatic trial.

Trained members of the research team will identify eligible participants utilising the scheduling lists for the relevant surgical procedures, these can be easily generated at each site. Furthermore, when patients are being scheduled for intervention, potential participants will be informed about the trial by the clinician involved in listing them for their procedure.

The study will be advertised by posters and leaflets placed in clinical areas and on social media and websites.

Members of the research team will contact potential participants and inquire if they would be willing to be involved in clinical research and interested in receiving the Participant Information Sheet (via post or email). This will be supplemented by information available on the trial website, including information videos and infographics.

Prior to contact, a medical records review will be undertaken to ensure eligibility.

The reasons for non-inclusion will be logged pseudoanonymously along with a minimum data set of age, gender and reason for exclusion. The pseudoanonymised

screening logs will be transferred to the Trial Coordinating Centre for the purposes of monitoring recruitment.

NIHR INCLUDE Guidance (31) was thoroughly consulted during development of the GRACE trial. The eligibility criteria and recruitment strategy have been designed to minimise potential underserving of patient groups. The need for therapeutic anticoagulation or having a thrombophilia, the main exclusion criteria, are not known to be associated with underserving of any groups by demographic, social, or health status factors with exception of atrial fibrillation (an indication for anticoagulation) having a higher prevalence with increasing age (33). However, we do not believe that this increasing prevalence will be sufficient to cause underserving of this population (33) given the increasing prevalence of osteoarthritis with age (the main clinical indication for hip and knee replacement) and malignancy (clinical indication for colectomy, nephrectomy, radical hysterectomy).

To ensure the trial recruits participants in which their first language is not English, we have costed for professional translation of the participant information sheet and informed consent documentation.

On a broader level, all hospitals will be included with varying levels of VTE risk assessment compliance rates to reflect real-world practice. We have also reviewed UK Index of Multiple Deprivation and will ensure recruitment sites will be representative.

Hence, we believe that the trial has been designed and with the aim to deliver it in a way that is acceptable and feasible to a broad (and representative) range of people who will be undergoing surgical procedures that require extended duration thromboprophylaxis.

5.1.1. RANDOMISATION

The secure web-based randomisation element of the Research Electronic Data Capture (REDCap) database will be used to randomly allocate consenting participants. Trained staff, who are listed on the delegation log, will confirm eligibility before proceeding to randomisation. The randomisation result will be available immediately.

Stratification will be built into this randomisation element by centre and type of surgery.

Individual participants will be randomised in a 1:1 fashion to one of two thromboprophylaxis strategies:
1. EDPTP* in addition to GCS (control arm), or
2. EDPTP alone (intervention arm)

*EDPTP includes any anti-thrombotic agent prescribed at a prophylactic dose for prevention of VTE, including low-molecular weight heparin (LMWH), Directly acting Oral AntiCoagulants (DOACs), or antiplatelet therapy.

5.1.2. INTERVENTION

Participants randomised to the intervention arm will receive EDPTP alone (no GCS during the admission).

Extended duration pharmacological thromboprophylaxis is the practice of prescribing the thromboprophylaxis for a duration after hospital discharge. Prevention of DVT is a licenced indication and recommended by NICE guidelines [NG89] for several Low molecular weight heparins (LMWH) such as Tinzaparin, Enoxaparin, or Direct oral anticoagulants (DOACs) such as Rivaroxaban, Apixaban. Aspirin is an antiplatelet medication that reduces platelet function with a variety of clinical indications. The use of aspirin as DVT prophylaxis in orthopaedic surgery is also recommended by NICE [NG89](17). Prophylactic-dose thromboprophylaxis is prescribed at a lower dose in

comparison to clinical indications for therapeutic anticoagulation, such as in the treatment of diagnosed DVT or PE.

5.1.3. CONTROL

Participants randomised to the control arm will receive both EDPTP post discharge and GCS for a period of time as per locally policy which may vary between Trusts.

GCS are elastic stockings worn on the lower limbs, often referred to by one of the brand names ThromboEmbolic Deterrent (TED®) stockings. They provide low pressure compression with the intended benefit to reduce the risk of VTE. Either below-knee or above-knee compression stockings may be used. The duration of stocking use should be as per local policy for example, until ambulant, discharge or post discharge.

The range of EDPTP available for the EDPTP and GCS stockings (the control group) will be the same as for the EDPTP alone group. As above agents commonly used include LMWH e.g., Tinzaparin, Enoxaparin, or DOACs e.g., Rivaroxaban, Apixaban. Prophylactic-dose thromboprophylaxis is prescribed at a lower dose in comparison to clinical indications for therapeutic anticoagulation, such as in the treatment of diagnosed DVT or PE. Extended duration pharmacological thromboprophylaxis is the practice of prescribing the thromboprophylaxis for a duration after hospital discharge. Prevention of DVT is a licenced indication for several LMWH and DOACs and recommended by NICE guidelines [NG89]. Aspirin is an antiplatelet medication that reduces platelet function with a variety of clinical indications. The use of aspirin as DVT prophylaxis in orthopaedic surgery is also recommended by NICE [NG89](17).

5.2 INFORMED CONSENT

Informed consent for enrolment will be ascertained from potential participants utilising either of the following approaches:

1. Prior to the day of procedure, through in person pre-assessment clinics or remotely utilising online meetings or telephone consultation.
2. On the day of, but prior to, their procedure through in person consultation.

Participants will have already received the Participant Information Sheet, and the Informed Consent form for reference, ahead of being approached for consent. Trained members of the research team will gain informed consent from participants.

Consent will be obtained in writing, verbally or electronically via econsent.

Any interested participants wishing to econsent will be emailed a link to the econsent form within the REDCAP database where they will be able to provide consent. RECAP provides an audit trail and signatures are attributed to the person completing this. The participant will be sent a copy of their consent form via email.

Participants who prefer to provide consent via telephone will be sent the information sheet and consent form for review via email. The research nurse/practitioner will then read off each statement on the consent form and ask the participant to confirm agreement with each statement. The researcher should initial the boxes with a note indicating that subjects had agreed to each point on the ICF. The research nurse/practitioner will then sign-off on the consent form to state that consent has been provided verbally over the telephone. The participant will be sent a copy of their consent form via email/post.

Prior to the surgical procedure, all participants will be provided with a leaflet which explains the signs and symptoms of developing a blood clot. Although VTE outcome will be assessed at 7, 21 to 35 days and 90-days post-procedure, participants will be advised to visit the emergency department if they suspect they have developed a blood clot (and not to wait for the study team to make contact).

Prior to the procedure, the following information will be collected from the patient and medical records:

- Baseline demographic information
- Name of surgical procedure
- Previous medical history and current medication

5.3 FOLLOW-UP

Participants will be contacted by the central study team at days 7, between 21 and 35 days, and 90 days to obtain follow up data. The follow-up will be conducted either by telephone, SMS, web or written depending on participant preference.

Participants will undergo a bilateral full lower limb (groin to ankle) deep venous thrombosis scan at 21- 35 days post-intervention to identify asymptomatic DVT, this is timed to capture the peak onset of events which is at 3 weeks (34). Reasonable travel expenses will be reimbursed for this visit.

Day 7 post procedure

Day 7 post procedure (data collected via telephone or online survey [link to survey sent via email or SMS])

- VTE outcome (participants will be asked to report on whether or not they have been diagnosed with a DVT or PE within the past 7-days)
- Adverse events associated with GCS will be captured (for those enrolled in the control arm)
- Participant reported adherence to GCS (for those enrolled in the control arm)
- Adherence to EDPTP

Day 21 to 35 post procedure

Day 21 to 35 post procedure (data collected via telephone or online survey [link to survey sent via email or SMS])

- VTE outcome (participants will be asked to report on whether or not they have been diagnosed with a DVT or PE since last contact)
- Adverse events associated with GCS will be captured (for those enrolled in the control arm)
- Participant reported adherence to GCS (for those enrolled in the control arm)
- Adherence to EDPTP
- Participants will also undergo a bilateral full lower limb (groin to ankle) deep venous thrombosis scan at 21- 35 days post-intervention to identify asymptomatic DVT

Day 90 post procedure

Day 90 post procedure (data collected via telephone or online survey [link to survey sent via email or SMS])

- VTE outcome (participants will be asked to report on whether or not they have been diagnosed with a DVT or PE since last contact)
- Adverse events associated with GCS will be captured (for those enrolled in the control arm)
- Participant reported adherence (for those enrolled in the control arm)
- Adherence to EDPTP

If the patient reports a VTE within the trial period that was not captured on the 21-to-35-day ultrasound we will request verification that imaging has confirmed the diagnosis.

Mortality within the 90-day follow-up period will also be captured. Follow-up data will be assessed blindly.

As this is a pragmatic trial, we will look to measure adherence and ensure it is reasonable but not attempt to introduce new interventions to increase adherence above what would be reasonably expected. We consulted the patient representatives, who have lived experience of wearing GCS, to establish how best to monitor adherence without unduly promoting increased adherence within the trial. Telephone consultation supplemented with remote messages by smart phone with electronic response to establish adherence was preferred. This was also reflected in wider PPI undertaken for other VTE prevention studies.

Any incidental findings identified during the study from the lower limb VTE ultrasound or self reported follow-up will be reported the clinical care team for any required treatment and follow-up.

5.4 END OF STUDY

The end of the study is defined as the last patient last visit.

6. STATISTICS AND DATA ANALYSIS

6.1 Sample size calculation

We have undertaken the most contemporary evidence synthesis, including data from 27 trial arms. Across 10,187 participants, EDPTP alone was associated with a DVT rate of 10.97%. With respect to EDPTP in addition to GCS, pooling 651 participants, the rate of DVT was 7.37%. The rate of PE in the literature is low, 0.05–0.11% and hence is unlikely to influence the sample size. These estimates are based largely on orthopaedic studies, 17 of the 27 study arms. The rate of DVT post major abdominal surgery and gynaecology oncology surgery tends towards to 5% (37, 38).
From search of NHS England Hospital Episode Statistics (HES) treatment episode we know ~80% of eligible participants will be undergoing orthopaedic joint replacement surgery, and hence the event rate will be closer to our summation analysis(26). The remaining 20% of cases will be from general and gynaecological cancer surgery. Hence, when accounting for these proportions, we anticipate the observed rate of VTE to lie close to 8%.

For 90% power at a 1-sided 2.5% level of significance, the study requires an unadjusted sample size of 7,736 to detect a non-inferiority margin of 2%, assuming the standard of care (EDPTP and GCS) DVT rate at 90 days is 8%. Following the same design as the NIHR/HTA funded GAPS study we will use a group sequential design with 2 scheduled interim analyses (at 40% and 65%, and a final analysis, at 100%, if not stopped early) to provide flexibility to stop early for overwhelming evidence of effectiveness or for futility. We will use an asymmetric 2-sided nonbinding Hwang-Shih-DeCani spending function, and this inflates the sample size to 8,264.

From GAPS, we know 4% of participants had a missing primary outcome, so we have adjusted the maximum expected sample size up to 8,608.

The non-inferiority margin has been set to 2%. Historically, VTE rates in the absence of treatment (i.e., no GCS or pharmacological thromboprophylaxis) were as high as 40% for joint replacement and 13% for abdominal and pelvic surgery. We have assumed an 8% VTE rate for standard of care (EDTPT in addition to GCS) and a 2% non-inferiority margin. Hence, the established benefit is 40-8=32%. We will preserve (32-2)/32 or 94% of the established benefit, with >2% representing a clinically meaningful difference by consensus from the expert opinion of the clinicians, and the patients.

6.2 Statistical analyses

Statistical analysis will be governed by a comprehensive SAP written by the study statistician and agreed by the independent Trial Steering Committee (TSC) and independent Data Monitoring Committee (iDMC) before any unblinded data is seen.

Main analysis will be according to intention-to-treat principle and will compare rates of VTE at 90 days, adjusting for any pre-specified strongly prognostic baseline covariates using a mixed effects logistic regression with study site as a random effect and pre-specified baseline covariates strongly related to outcome being used to adjust the estimated treatment effect. Primary outcome will be VTE at any time up to 90 days. The findings will be assessed for robustness against any missing data, first using multiple imputation assuming this data is missing at random and, if appropriate and the data permits, further sensitivity analyses will be attempted under any plausible missing data mechanisms not missing at random. In particular, since for non-inferiority designs, in which the null hypothesis is that the randomised groups differ, the intention-to-treat analysis may not be the most conservative, and so we will conduct a suitably specified Per Protocol analysis (defined by adherence to study protocol) as a supporting analysis to the main ITT analysis. We will also include a time to event specification (a Fine & Gray) model of the primary endpoint to check on the influence of any competing risk of death (expected to be a rare event at ~1% overall). Secondary

outcomes will be analysed in a similar fashion with generalised linear models appropriate to the distribution of the outcome. Safety data will be summarised descriptively. An additional sensitivity analysis of NHS-only patients (including patients referred by their GPs to non-NHS sites) will be conducted to ensure generalisability to a NHS population.

6.3 Subgroup analyses

We will perform the following sub-group analyses of the primary outcome, and test for sub-group interactions if appropriate:

Predefined sub-group analyses (primary outcome) include:

- Sex (male, female)
- Age
- BMI
- Smoking status (ex-smoker, current smoker, never smoker)
- Type of anaesthesia (regional, general)

The study is not formally powered for these subgroups analyses and will be exploratory.

6.4 Interim analysis

We have scheduled two unequally spaced interim analyses (at 40% and 65%) and at 100% (as a final analysis if the study is not stopped early at any of the interim analyses) to provide flexibility to stop early for overwhelming evidence of effectiveness or for futility. The z-value thresholds for futility will be 0.05 and 0.86, and for ‘effectiveness’ 2.90 and 2.59, with the threshold for non-inferiority at the final analysis (if reached) 2.00. Recruitment will continue while the interim data is analysed.

Full details of the stopping boundaries and analysis will be included in the SAP, which the iDMC will approve prior to seeing unblinded data. This interim analysis will be presented by the unblinded statistician at ECTU to the iDMC, who will include an

independent statistician. The unblinded ECTU statistician would have no other role in the study while it was ongoing. The stopping rules are statistically non-binding. The iDMC may recommend early stopping of the study if the boundaries are crossed. They would make a recommendation to the independent Trial Steering Committee (TSC) who may or may not endorse that recommendation. Note also that the trial can stop at any time for safety, if there is an excess of events in the intervention group that is considered to generate avoidable harm – and this would be a decision not based on any statistical criterion and taken by the iDMC and endorsed by the TSC.

For a non-inferiority design, futility corresponds to showing one or the other of the randomised interventions is superior; whereas ‘evidence of effectiveness’ is showing early that the drugs alone are indeed non-inferior to the combination.

7. ADVERSE EVENTS

7.1. DEFINITIONS

Adverse Event (AE): any untoward medical occurrence in a patient or clinical study subject.

Serious Adverse Event (SAE): any untoward medical occurrence or effect that:

- **Results in death**
- **Is life-threatening** – *refers to an event in which the subject was at risk of death at the time of the event; it does not refer to an event which hypothetically might have caused death if it were more severe*
- **Requires hospitalisation, or prolongation of existing inpatients’ hospitalisation**
- **Results in persistent or significant disability or incapacity**
- **Is a congenital anomaly or birth defect**

Medical judgement should be exercised in deciding whether an AE is serious in other situations. Important AEs that are not immediately life-threatening or do not result in death or hospitalisation but may jeopardise the subject or may require intervention to

prevent one of the other outcomes listed in the definition above, should also be considered serious.

7.2. REPORTING PROCEDURES

For the purposes of the study, only AEs related to the study intervention (graduated compression stockings) will be recorded. This information will be collected at 7-days, between 21 and 35 days and at 90 days.

Safety monitoring includes any adverse events with GCS or bleeding events with the standard of care pharmacological thromboprophylaxis. Major bleeding will be defined as per the International Society on Thrombosis and Haemostasis definition (36).

Participants who report a treatment related AE which requires further investigation/follow-up will be advised to consult with their GP/relevant clinical team where necessary. Depending on the nature of the event the reporting procedures below should be followed. Any questions concerning adverse event reporting should be directed to the Chief Investigator in the first instance.

7.2.1 Non serious AEs

Only AEs related to the study intervention (graduated compression stockings) will be recorded.

7.2.2 Serious AEs

An SAE form should be completed and emailed to the Chief Investigator within 24 hours. However, hospitalisations for elective treatment of a pre-existing condition do not need reporting as SAEs.

All SAEs should be reported to the Research Ethics Committee where in the opinion of the Chief Investigator, the event was:

- ‘related’, ie resulted from the administration of any of the research procedures; and
- ‘unexpected’, ie an event that is not listed in the protocol as an expected occurrence

Reports of related and unexpected SAEs should be submitted within 15 days of the Chief Investigator becoming aware of the event, using the NRES SAE form for non-IMP studies. The Chief Investigator must also notify the Sponsor of all related and unexpected SAEs.

Local investigators should report any SAEs as required by their Local Research Ethics Committee, Sponsor and/or Research & Development Office.

Contact details for reporting SAEs
RGIT@imperial.ac.uk
Please send SAE forms to: gracetrial@imperial.ac.uk
Tel: +44 (0)203 311 7371 (Mon to Fri 09.00 – 17.00)

8. REGULATORY ISSUES

8.1. ETHICS APPROVAL

The Study Coordination Centre has obtained approval from the Wales 3 Research Ethics Committee (REC) and Health Research Authority (HRA). The study must also receive confirmation of capacity and capability from each participating NHS Trust before accepting participants into the study or any research activity is carried out. The study will be conducted in accordance with the recommendations for physicians involved in research on human subjects adopted by the 18th World Medical Assembly, Helsinki 1964 and later revisions.

8.2. CONSENT

Consent to enter the study will be sought from each participant only after a full explanation has been given, an information leaflet offered and time allowed for consideration. Consent will be obtained in written, verbal or electronic form. The right of the participant to refuse to participate without providing any reason will be respected. After the participant has entered the study, the clinician remains free to give alternative treatment to that specified in the protocol at any stage if he/she feels it is in the participant's best interest, but the reasons for doing so should be recorded. In these cases the participants remain within the study for the purposes of follow-up and data analysis. All participants are free to withdraw at any time from the protocol treatment without giving reasons and without prejudicing further treatment.

8.3. CONFIDENTIALITY

The Chief Investigator will preserve the confidentiality of participants taking part in the study and is registered under the Data Protection Act.

As follow-ups will be performed by the coordinating centre (Imperial College London), patient identifiable data (name, address, email address and contact telephone number[s]) will be stored on the REDCap database. This identifiable data will only be accessible by researchers at the local site (who will enter the data onto REDCap in the first place) and by the blinded assessors based at the coordinating centre who are responsible for conducting the follow-ups.

The trial manager will only have access to pseudonymised data on REDCap.

The investigator shall permit direct access to subjects' records and source document for the purposes of monitoring, auditing, or inspection by the Sponsor, authorised representatives of the Sponsor and RECs.

Data will be transferred to The University of Edinburgh for the purposes of statistical analysis.

8.4. INDEMNITY

Imperial College London holds negligent harm and non-negligent harm insurance policies which apply to this study.

8.5. SPONSOR

Imperial College London will act as the main Sponsor for this study. Delegated responsibilities will be assigned to the sites taking part in this study.

8.6. FUNDING

The National Institute for Health Research (NIHR) are funding this study. Participants will be reimbursed for reasonable travel costs for the VTE scan visit. Individual researchers will not receive any personal payment over and above normal salary, or any other benefits or incentives, for taking part in this research

8.7. AUDITS

The study may be subject to audit by Imperial College London under their remit as sponsor and other regulatory bodies to ensure adherence to GCP and the UK Policy Frame Work for Health and Social Care Research.

9. DATA MANAGEMENT

9.1. DATA COLLECTION

The source data is captured in the source worksheets which will then be transcribed into the eCRF (Redcap). Details of procedures for eCRFcompletion will be provided in a study manual.

A formal Data Management Plan will be constructed to describe the procedures involved in the data management activities and processes for the study so that it is managed and maintained in accordance with ICH-Good Clinical Practice (GCP) guidelines, local Research Governance and Integrity Team (RGIT) standard operating procedures (SOPs), appropriate regulatory requirements and the study protocol.

Trial data will be generated from the DVT scan reports and completion of case report forms undertaken by telephone consultation, online survey, and remote text messages. Source data can be in the form of, but not limited to, patient medical records and study-specific case report forms. This will be stored locally at the study centres in a secure space accessed only by authorised personnel. Data will be manually entered into the database by dedicated staff at the study centres and this will be verified by the Trial Manager.

The software model for the data entry processes and monitoring will be REDCap, which is a secure, validated, web-based software platform providing an intuitive interface for validated data capture, audit trails for tracking data manipulation and export procedures, automated export procedures for seamless data downloads to

common statistical packages, and procedures for data integration and interoperability with external sources. Edinburgh Clinical Trials Unit will be responsible for randomisation, the database build and system validation and REDCap will be responsible for hosting the data. During the running of the trial, Reporting Data Extracts (RDE) will be provided for safety reporting, independent data monitoring committee meetings, interim and final analysis, and other reporting periods as outlined in the Statistical Analysis Plan (SAP).

Only pseudo-anonymised data will be shared under the terms of the consent forms and will only be available to users under a data-sharing request. Requests made outside the host organisation will be made under a data sharing agreement (signed by the CI/Trial Steering Committee) that provides the following: (1) A commitment to using the data only for research purposes and not to identify any individual participant; (2) A commitment to securing the data using appropriate computer technology; and (3) A commitment to destroying or returning the data after analyses. A record of all access to data will be maintained by Data Management on the Data Sharing Log. The study database will be locked before the final analysis. After analysis, all study data will be stored and archived in accordance with the RGIT SOPs.

All protocol deviations and protocol violations will be reported via the eCRF/CRF and reviewed by the Chief Investigator. Protocol violations will be reported to the Sponsor.

An assessment of whether the protocol deviation/violation constitutes a serious breach will be made.

A serious breach is defined as:
A breach of the conditions and principles of GCP in connection with a trial or the trial protocol, which is likely to affect to a significant degree:

- The safety or physical or mental integrity of the UK trial participants; or
- The overall scientific value of the trial

The Sponsor will be notified within 24 hours of identifying a likely Serious Breach. If a decision is made that the incident constitutes a Serious Breach, this will be reported to the REC within 7 days of becoming aware of the serious breach.

9.2. ARCHIVING

Source data stored at study sites will be archived locally as per local SOPs. Data and all appropriate documentation will be stored for a minimum of 10 years after the completion of the study, including the follow-up period.

10. STUDY MANAGEMENT

The day-to-day management of the study will be co-ordinated through Imperial College London.

11. PUBLICATION POLICY

The following outputs are anticipated to arise from the GRACE trial:

- Publications in peer reviewed journals (including the protocol paper and main trial analysis)
- The NICE guidelines aiming to prevent VTE “Venous thromboembolism in over 16s: reducing the risk of hospital-acquired deep vein thrombosis or pulmonary embolism [NG89]” will be subsequently updated
- Cost-effectiveness information to guide clinical commissioning groups and NICE
- Updated systematic review of literature and meta-analysis
- An understanding of the safety of GCS and subsequent quality of life
- Presentation at international academic conferences including European and American vascular, venous, general surgery and haematology societies

- The results of the trial will be emailed to participants and published through patient groups such as Thrombosis UK.
- Dissemination of results to the wider public through social media streams
- If GCS are found to ineffective, this could prompt a wider effect on the design and application of graduated compression stocking devices within the healthcare setting

12. REFERENCES

1. NHS Digital. 5.1 deaths from venous thromboembolism (VTe) related events within 90 days post discharge from hospital. 2019.
2. Cohen AT, Agnelli G, Anderson FA, et al. Venous thromboembolism (VTE) in Europe. Thrombosis and haemostasis. 2007;98(10):756-64.
3. Committee HoCH. The prevention of venous thromboembolism in hospitalised patients. London: The Stationery Office. 2005;20005.
4. Brækkan SK, Grosse SD, Okoroh EM, et al. Venous thromboembolism and subsequent permanent work-related disability. Journal of Thrombosis and Haemostasis. 2016;14(10):1978-87.
5. Kahn SR, Shapiro S, Wells PS, et al. Compression stockings to prevent post-thrombotic syndrome: a randomised placebo-controlled trial. The Lancet. 2014;383(9920):880-8.
6. Eklof B, Perrin M, Delis KT, Rutherford RB, Gloviczki P. Updated terminology of chronic venous disorders: the VEIN-TERM transatlantic interdisciplinary consensus document. Journal of vascular surgery. 2009;49(2):498-501.
7. Kahn S, M'lan C, Lamping D, Kurz X, Berard A, Abenhaim for the Veines Study Group L. The influence of venous thromboembolism on quality of life and severity of chronic venous disease. Journal of thrombosis and haemostasis. 2004;2(12):2146-51.
8. Ware JE, Kosinski M, Keller S. SF-36 physical and mental health summary scales: a user's manual: Health Assessment Lab.; 1994.
9. Kahn S, Shbaklo H, Lamping D, et al. Determinants of health-related quality of life during the 2 years following deep vein thrombosis. Journal of thrombosis and haemostasis. 2008;6(7):1105-12.
10. Noble S, Lewis R, Whithers J, Lewis S, Bennett P. Long-term psychological consequences of symptomatic pulmonary embolism: a qualitative study. BMJ open. 2014;4(4):e004561.
11. Barco S, Russo M, Vicaut E, et al. Incomplete echocardiographic recovery at 6 months predicts long-term sequelae after intermediate-risk pulmonary embolism. A post-hoc analysis of the Pulmonary Embolism Thrombolysis (PEITHO) trial. Clinical Research in Cardiology. 2019;108(7):772-8.
12. Guérin L, Couturaud F, Parent F, et al. Prevalence of chronic thromboembolic pulmonary hypertension after acute pulmonary embolism. Thrombosis and haemostasis. 2014;112(09):598-605.
13. Ende-Verhaar YM, Cannegieter SC, Noordegraaf AV, et al. Incidence of chronic thromboembolic pulmonary hypertension after acute pulmonary embolism: a contemporary view of the published literature. European Respiratory Journal. 2017;49(2).
14. Heijkoop B, Nadi S, Spernat D, Kiroff G. Extended versus inpatient thromboprophylaxis with heparins following major open abdominopelvic surgery for malignancy: a systematic review of efficacy and safety. Perioperative Medicine. 2020;9(1):1-8.
15. Anderson Jr FA, Spencer FA. Risk factors for venous thromboembolism. Circulation. 2003;107(23_suppl_1):I-9-I-16.
16. BARNES RW, BRAND RA, CLARKE W, HARTLEY N, HOAK JC. Efficacy of graded-compression antiembolism stockings in patients undergoing total hip arthroplasty. Clinical Orthopaedics and Related Research®. 1978(132):61-7.

17. UK NGC. Venous thromboembolism in over 16s: reducing the risk of hospital-acquired deep vein thrombosis or pulmonary embolism. 2018.
18. Shalhoub J, Lawton R, Hudson J, et al. Graduated compression stockings as adjuvant to pharmaco-thromboprophylaxis in elective surgical patients (GAPS study): randomised controlled trial. bmj. 2020;369.
19. Sachdeva A, Dalton M, Lees T. Graduated compression stockings for prevention of deep vein thrombosis. Cochrane Database of Systematic Reviews. 2018(11).
20. Chin P, Amin M, Yang K, Yeo S, Lo N. Thromboembolic prophylaxis for total knee arthroplasty in Asian patients: a randomised controlled trial. Journal of orthopaedic surgery. 2009;17(1):1-5.
21. Dennis M, Sandercock PA, Reid J, Graham C, Murray G, Venables G, et al. Effectiveness of thigh-length graduated compression stockings to reduce the risk of deep vein thrombosis after stroke (CLOTS trial 1): a multicentre, randomised controlled trial. Lancet. 2009;373(9679):1958-65.
22. Cohen A, Skinner J, Warwick D, Brenkel I. The use of graduated compression stockings in association with fondaparinux in surgery of the hip: a multicentre, multinational, randomised, open-label, parallel-group comparative study. The Journal of Bone and Joint Surgery British volume. 2007;89(7):887-92.
23. Page MJ, McKenzie JE, Bossuyt PM, et al. The PRISMA 2020 statement: an updated guideline for reporting systematic reviews. Systematic reviews. 2021;10(1):1-11.
24. (NICE) NIfHaCE. Venous thromboembolism in adults, Quality standard [QS201] 2021 [cited 2022 16/12/2022]. Available from: https://www.nice.org.uk/guidance/qs201.
25. Roberts LN, Durkin M, Arya R. Annotation: developing a national programme for VTE prevention. British Journal of Haematology. 2017;178(1):162-70.
26. NHS Digital. Hospital Episode Statistics (HES), Hospital Admitted Patient Care Activity 2022 [cited 2022 03/12/2022]. Available from: https://digital.nhs.uk/data-and-information/data-tools-and-services/data-services/hospital-episode-statistics#hes-publications.
27. Unit PSSR. Unit Costs of Health and Social Care 2019. 2019.
28. Ziaja D, Kocełak P, Chudek J, Ziaja K. Compliance with compression stockings in patients with chronic venous disorders. Phlebology. 2011;26(8):353-60.
29. Kuroiwa M, Takahira N, Ujihashi Y, Miida K, Arai Y, Kawatani H. Reduction in the soles of graduated compression stockings prevents falls without reducing the preventive effect for venous stasis. Thrombosis research. 2015;135(5):877-81.
30. White RH, Zhou H, Romano PS. Incidence of symptomatic venous thromboembolism after different elective or urgent surgical procedures. Thrombosis and haemostasis. 2003;90(09):446-55.
31. Merkow RP, Bilimoria KY, McCarter MD, et al. Post-discharge venous thromboembolism after cancer surgery: extending the case for extended prophylaxis. Annals of surgery. 2011;254(1):131-7.
32. Research NIfHaC. Improving inclusion of under-served groups in clinical research: Guidance from the NIHR-INCLUDE project. UK: NIHR.; 2020 16/12/2022.
33. Zoni-Berisso M, Lercari F, Carazza T, Domenicucci S. Epidemiology of atrial fibrillation: European perspective. Clinical epidemiology. 2014;6:213.

34. Sweetland S, Green J, Liu B, et al. Duration and magnitude of the postoperative risk of venous thromboembolism in middle aged women: prospective cohort study. Bmj. 2009;339.
35. Williamson PR, Altman DG, Bagley H, Barnes KL, Blazeby JM, Brookes ST, et al. The COMET Handbook: version 1.0. Trials. 2017;18(3):280.
36. Schulman S, Angerås U, Bergqvist D, et al. Definition of major bleeding in clinical investigations of antihemostatic medicinal products in surgical patients. Journal of Thrombosis and Haemostasis. 2010;8(1):202-4.
37. Ibrahim E, Norris LA, Saadeh FA. Update on extended prophylaxis for venous thromboembolism following surgery for gynaecological cancers. Thrombosis Update. 2021;2:100038.
38. Rausa E, Kelly ME, Asti E, et al. Extended versus conventional thromboprophylaxis after major abdominal and pelvic surgery: Systematic review and meta-analysis of randomized clinical trials. Surgery. 2018;164(6):1234-40.
39. Service NDR. The National Cancer Registration and Analysis Service 2022 [updated 08/2022. Available from: http://www.ncin.org.uk/home.

Appendix 1. Summary of investigations, treatment and assessments

Assessment	Days of assessment				
	Pre-surgical procedure	0 (Day of procedure)	7	21 to 35	90
Inclusion/Exclusion	X	X			
Informed consent[1]	X				
Screening assessments	X				
Demographics[1]		X			
Medical history (including concurrent medications)[1]		X	X	X	
Provision of leaflet explaining the signs and symptoms of developing a blood clot	X				
Provision of stockings*		X			
VTE outcome (self-reported)			X	X	X
A full lower limb deep venous thrombosis scan				X	
Adverse Events Assessment*			X	X	X
Adherence to GCS*			X	X	X
Adherence to EDPTP			X	X	X

*Patients randomised to the control arm only
[1]Can be collected at any point up to the 7-day follow-up, including prior to the day of procedure (i.e. at the preassessment stage) or on the day of procedure
