# Supplementary material for: GRACE: protocol for a UK, secondary care, multicentre, assessor-blinded randomised controlled trial with a non-inferiority comparison to evaluate graduated compression stockings as an adjunct to extended duration pharmacological thromboprophylaxis for venous thromboembolism prevention
Source: BMJ Open. 2025 Jul 6;15(7):e095482. doi: 10.1136/bmjopen-2024-095482 (PMC12230952; doi:10.1136/bmjopen-2024-095482)
Supplement: online supplemental file 2 [file bmjopen-15-7-s002.pdf]

Supplemental Material

Study protocol for Graduated Compression stocking as an adjunct to Extended duration pharmacological thromboprophylaxis for venous thromboembolism prevention

(The GRACE multi-centre randomised controlled trial)

Appendix 1: Patient information sheet/ informed consent……………………………pg1-19

Appendix 1: Patient information sheet/ informed consent

Imperial College London

GRACE

GRAduated Compression stocking as an adjunct to Extended duration pharmacological thromboprophylaxis for venous thromboembolism prevention (GRACE Trial)

ISRCTN11667770

IRAS: 333539

Sponsor Reference: 22HH7932

Acknowledgement

This project is funded by a National Institute for Health Research, Health Technology Assessment programme grant, which is funded by the National Institute for Health Research (NIHR)

Disclaimer

The views expressed in this publication are those of the author(s) and not necessarily those of the MRC, NHS, NIHR or the Department of Health.

GRACE Patient Information Sheet V2.0, 15/05/2024
Approved by Wales REC 3 on 20/12/2023

PATIENT INFORMATION SHEET DOCUMENT

You have been invited to take part in a research study called GRACE. Before you decide whether to accept, we would like to explain why the research is being carried out and what it will involve.

Please read this information carefully and discuss it with others if you wish. Ask us if anything is unclear, or if you would like more information.

- *Part 1 tells you the purpose of this study and what will happen to you if you take part.*
- *Part 2 gives you more detailed information about the conduct of the study.*

Please take your time to decide whether or not you wish to take part.

Thank you for reading this information sheet.

Part 1

What is the purpose of the study?

Hospital acquired thrombosis describes blood clots that form in the legs and lungs after someone is treated in hospital. A clot in the leg can cause swelling, pain and other problems. If a clot in the leg breaks off and travels to the lungs, it may be life threatening. Having surgery increases the risk of developing blood clots. Blood clots in the legs and/or lungs are collectively known as venous thromboembolism (VTE).

Doctors have known about the risks of patients developing blood clots after operations for many years and use two main ways to prevent this:

- Thinning the blood with injections / tablets, and
- Wearing elastic stockings to help stop blood sitting in the leg veins where it can clot.

All participants who undergo surgery that puts them at higher risk of developing VTE require the blood thinners to be taken post discharge from hospital. Currently, some hospitals provide elastic stockings in addition to blood thinners but other hospital do not. There are no good quality research studies to show if the addition of elastic stockings reduces the risk of VTEs in patients taking blood thinners after discharge from hospital. This study is being done to find out if this is true.

Why have I been chosen?

You have been invited to consider this study because you are scheduled to undergo a procedure in hospital that puts you at higher risk of developing VTE (a surgical procedure which means you will be prescribed a course of blood thinners post discharge from hospital). We hope that 8606 people like you from across the UK will take part in this study.

Do I have to take part?

No, participation in this study is entirely voluntary. If you do decide to take part you will be given this information sheet to keep. You will be asked to sign a consent form, but you are still free to withdraw at any time and without giving a reason. If you decide not to take part in the study, we will not contact you after your procedure. Your doctor will be happy to talk through how they will treat you outside of the study. You do not have to give a reason for not taking part and your treatment and care will not be affected in any way.

What will happen to me if I take part?

If you agree to enter the trial, you will be allocated by computer to either the 'elastic stockings plus blood thinner' group or the 'blood thinner alone' group. There is an equal chance of either treatment being the one you will receive. Neither your doctor nor you will choose which treatment you receive. In this way, a fair comparison between the two groups can be made.

If you choose to be involved in the research, your participation will last 3-months from entry. After you have undergone your surgery, we will contact you at three time points (7 days, between 21 and 35 days and at 90 days later) to ask you some short questions about your health and how often you have worn your stockings and taken your blood thinners, this should only take a few minutes. These questions can be answered via telephone or an online survey (the link to this survey will be sent via email or text message). Your contact details will be stored on the study database. Members of the research team from Imperial College London will have access to your contact details so that they can contact you.

You will also be invited back to the hospital between 21 and 35 days to have an additional scan of the veins in your legs to detect any blood clots that may have developed.

First contact

You will first be asked to provide your consent to confirm that you would like to be included in the study. Your consent can be provided in a number of ways – it can be provided electronically (e.g. by confirming your participation online), verbally (e.g. by informing the research team over the telephone that you would like to take part) or in writing (by signing a 'consent form'). Regardless of how your consent has been provided, you will be provided with a copy of the 'consent form' held by the research team.

We will then collect some information about your medical history and current medical conditions to check that you are able to take part.

Prior to you entering the study, you will be provided with a leaflet which explains how to recognise the signs and symptoms of a blood clot. Although rare, a blood clot can occur in anyone and without any warning signs or symptoms. They most commonly occur in the limbs (called deep vein thrombosis; DVT), or in the lungs (a pulmonary embolism; PE). Signs and symptoms of a DVT can include leg pain or/or swelling. Signs and symptoms of a PE can include shortness of breath, difficulty breathing and chest pain.

If you suspect that you have developed a blood clot at any point during your time in the trial, it is very important that you seek help from a healthcare professional (e.g., by visiting the emergency department or contacting 999 or 111) immediately. Please do not wait to be contacted by a member of the study team.

Second contact: 7-days after procedure

Seven-days after you have undergone your surgical procedure, we will contact you remotely (i.e. via telephone, email/text message [containing a link to an online survey]) and ask you a few short questions to see how you have been getting on.

We will ask you to confirm whether or not you have been diagnosed with a blood clot in the legs (also known as a DVT) or a blood clot in the lungs (also known as a PE). We will also ask you if you're still taking blood thinners.
Finally, if you received elastic stockings whilst in hospital, we will ask you to report on any side effects you think you experienced as a result of wearing these and how long you wore them.

Third contact: between 21 and 35 days after procedure
We will contact you again remotely (i.e., via telephone, email/text message [containing a link to an online survey]) and ask you the same questions as the previous visit.
You will also be invited back to the hospital during this time for an additional scan of the veins in your legs to detect any blood clots that may have developed.

Final contact: 90-days after procedure
Approximately 90-days after you have undergone your surgical procedure, we will contact you again remotely (i.e. via telephone, email/text message [containing a link to an online survey]) and ask you the same questions as the previous visit.

You may be withdrawn from the study if the doctor feels it is best for you not to participate or if you do not comply with the requirements of the study.

What are the possible benefits of taking part?

We cannot promise the study will help you but the information gained from the study may help healthcare professionals make decisions in future as to whether elastic stockings are helpful for preventing blood clots in patients who are deemed higher risk of developing a blood clot.

Participants in both groups of the trial will be monitored closely for any complications of stockings and blood thinners, so that any complications can be detected and acted upon. Patients will have an extra non-invasive leg scan between 21 and 35 days after their operation. This will detect any blood clots in the legs that the patient did not know they had, so treatment for the blood clot can be started. Patients not entered into the study would not normally be offered this scan unless they showed symptoms.

What are the possible disadvantages and risks of taking part?

Side effects of elastic stockings are uncommon. Whilst we do not anticipate any specific side effects as a result of taking part in this trial, in rare circumstances, some patients may be allergic to the materials that are contained within the stockings. Furthermore, some people find the stockings uncomfortable to wear but this causes no lasting effects. Very rarely the compression stockings can cause abrasions of the skin. Lastly, we are uncertain if wearing stockings reduces the chances of a blood clot developing which is why we are organising this trial.

What happens when the research study stops?

The information from this study will be used to decide if elastic stockings are beneficial for the prevention of blood clots in patients who are required to take blood thinning medication post hospital discharge.

Will my taking part be kept confidential?

Yes, it will. If you decide to participate, the information collected about you will be handled strictly in accordance with the consent form that you have signed and also the 2018 Data Protection Act. Please refer to Part 2 for further details.

This completes Part 1 of the Information Sheet. If the Information in Part 1 has interested you and you are considering participation, please continue to read the additional information in Part 2 before making any decision.

Part 2

What if relevant new information becomes available?

Sometimes during the course of a research project, new information becomes available about a treatment that is being studied. If this happens, the research team will tell you about it and will discuss with you whether you want to continue in the study. If you decide to withdraw, the research team will make arrangements for your care to continue. If you decide to continue in the study you will be asked to sign an updated consent form.

What will happen if I don't want to carry on with the study?

You can decide to leave the study at any time. You do not need to give a reason. If you decide to leave the study, any data collected up until that time will remain on file and will be included in the final study analysis. If you do not wish for any further data to be collected about you, you should inform your clinical care team of this in order that no further follow up information is collected. In line with Good Clinical Practice guidelines, at the end of the study, your data will be securely archived. In this study, data will be archived for a minimum of 10 years after which arrangements for confidential destruction will be made.

What if something goes wrong?

A group of independent researchers (called the Data Monitoring Committee) will closely monitor the study. If there are any problems then they will be detected as soon as possible so that the study can be changed or stopped if necessary.

Imperial College London holds insurance policies which apply to this study. If you experience harm or injury as a result of taking part in this study, you will be eligible to claim compensation without having to prove that Imperial College is at fault. This does not affect your legal rights to seek compensation.

If you are harmed due to someone's negligence, then you may have grounds for legal action. Regardless of this, if you wish to complain, or have any concerns about any aspect of the way you have been treated during the course of this study then you should immediately inform the Investigator (Insert name and contact details). The normal National Health Service complaints mechanisms are also available to you. If you are not satisfied with the response, you may contact your local Patient Advice and Liaison Service (PALS) which offers confidential advice, support and information on health-related matters (insert contact details) (delete if non-NHS). You may also contact the Imperial AHSC Research Governance and Integrity office.

How will we use information about you?

Imperial College London is the sponsor for this study and will act as the Data Controller for this study. This means that we are responsible for looking after your information and using it appropriately. Imperial College London will keep your personal data for:

- 10 years after the study has finished in relation to data subject consent forms.
- 10 years after the study has completed in relation to primary research data.

  The study is expected to finish in April 2027. For more information / confirmation regarding the end date please contact the study team, see 'WHERE CAN YOU FIND OUT MORE ABOUT HOW YOUR INFORMATION IS USED' for contact information.

We will need to use information from you and from your medical records and possibly your GP for this research project.

This information will include your initials, NHS number, name, contact details including email address and telephone number.

People within the College and study team (see section sharing your information with others) will use this information to do the research or to check your records to make sure that research is being done properly and the information held (such as contact) details is accurate.

People who do not need to know who you are will not be able to see your name or contact details. Your data will have a code number instead. We will keep all information about you safe and secure. Some of your information will be sent to the University of Edinburgh in the United Kingdom. They must follow our rules about keeping your information safe.

Once we have finished the study, we will keep some of the data so we can check the results. We will write our reports in a way that no-one can work out that you took part in the study.

As a university we use personally-identifiable information to conduct research to improve health, care and services. As a publicly-funded organisation, we have to ensure that it is in the public interest when we use personally-identifiable information from people who have agreed to take part in research. This means that when you agree to take part in a research study, we will use your data in the ways needed to conduct and analyse the research study. Our legal basis for using your information under the General Data Protection Regulation (GDPR) and the Data Protection Act 2018, is as follows:

• Imperial College London - “performance of a task carried out in the public interest”); Health and care research should serve the public interest, which means that we have to demonstrate that our research serves the interests of society as a whole. We do this by following the UK Policy Framework for Health and Social Care Research.

Where special category personal information is involved (most commonly health data, biometric data and genetic data, racial and ethnic data etc.), Imperial College London rely on “scientific or historical research purposes or statistical purposes.

International Transfers

There may be a requirement to transfer information to countries outside the United Kingdom (for example, to a research partner, either within the European Economic Area (EEA) or to other countries outside the EEA. Where this information contains your personal data, Imperial College London will ensure that it is transferred in accordance with data protection legislation. If the data is transferred to a country which is not subject to a UK adequacy decision in respect of its data protection standards, Imperial College London will enter into a data sharing agreement with the recipient research partner that incorporates UK approved standard contractual clauses or utilise another transfer mechanism that safeguards how your personal data is processed.

Sharing your information with others

We will only share your personal data with certain third parties for the purposes referred to in this participant information sheet and by relying on the legal basis for processing your data as set out above.

Other Imperial College London employees (including staff involved directly with the research study or as part of certain secondary activities which may include support functions, internal audits, ensuring accuracy of contact details etc.), Imperial College London agents, contractors and service providers (for example, suppliers of printing and mailing services, email communication services or web services, or suppliers who help us carry out any of the activities described above).

Our third-party service providers are required to enter into data processing agreements with us. We only permit them to process your personal data for specified purposes and in accordance with our policies.

· The following Research Collaborators / Partners in the study: University of Edinburgh – with your permission, your data will be entered onto a secure database held at the University of Edinburgh, in accordance with the 2018 Data Protection Act. With your consent, your mobile phone number and email address will be entered into a secure database hosted by the University of Edinburgh. These contact details will be used to make contact for with you for your follow-ups. This organisation will also perform the statistical analysis for the study and will have access to all pseudoanonymised data.

Potential use of study data for future research

When you agree to take part in a research study, the information collected either as part of the study or in preparation for the study (such as contact details) may, if you consent, be provided to researchers running other research studies at Imperial College London and in other organisations which may be universities or organisations involved in research in this country or abroad. Your information will only be used to conduct research in accordance with legislation including the GDPR and the UK Policy Framework for Health and Social Care Research.

This information will not identify you and will not be combined with other information in a way that could identify you, used against you or used to make decisions about you.

What are your choices about how your information is used?

You can stop being part of the study at any time, without giving a reason, but we will keep information about you that we already have because some research using your data may have already taken place and this cannot be undone.

If you choose to stop taking part in the study, we would like to continue collecting information about your health from your hospital or your GP. If you do not want this to happen, tell us and we will stop. This will not affect any healthcare or support you may be receiving separately.

We need to manage your records in specific ways for the research to be reliable. This means that we won't be able to let you see or change the data we hold about you if this could affect the wider study or the accuracy of data collected.
If you agree to take part in this study, you will have the option to take part in future research using your data saved from this study.
Data may also be linked with appropriate national databases, including Hospital Episode Statistics (HES), as well as for longer term follow-up in the event the trial is extended.

Where can you find out more about how your information is used?
You can find out more about how we use your information

- at www.hra.nhs.uk/information-about-patients/
- by asking one of the research team
- by sending an email to gracetrial@imperial.ac.uk, or
- by ringing us on (0)203 311 7371.

Complaint
If you wish to raise a complaint about how we have handled your personal data, please contact the research team first by sending an email to gracetrial@imperial.ac.uk or by ringing us on 0203 3117371.

Following our response, if you are not satisfied please contact Imperial College London's Data Protection Officer via email at dpo@imperial.ac.uk, via telephone on 020 7594 3502 and/or via post at Imperial College London, Data Protection Officer, Faculty Building Level 4, London SW7 2AZ.

If you remain unsatisfied with our response or believe we are processing your personal data in a way that is not lawful you can complain to the Information Commissioner's Office (ICO)- via www.ico.org.uk. Please note the ICO does recommend that you seek to resolve matters with the data controller (us) first before involving them.

Involvement of the General Practitioner (GP) / Family Doctor:
With your permission, your GP and other doctors involved in your clinical care will be kept informed of your participation in the study, but otherwise all information about you and your treatment will remain confidential.

What will happen if I lose mental capacity during the study period?
This is expected to be a very rare occurrence. If this did occur your doctor or carer will determine whether you should be withdrawn from the study. If you are withdrawn, any identifiable data already collected with consent will be retained and may be analysed, but no further data will be collected or any other research procedures carried out on or in relation to you.

What will happen to the results of the research study?
When the study is complete, we plan to inform participants of the results of the study by letter, email, newsletter, social media or publication on the trial website. We may ask patients if there are any other methods they would prefer. The results will be presented at conferences and published in a medical journal. No individual participants will be identified.

Who has organised, reviewed and funded the research and who will be supervising it?
This research has been supported by a National Institute for Health Research, Health Technology Assessment programme grant, which is funded by the National Institute for Health Research. The Sponsor of this study (Imperial College London) will pay your hospital to cover the costs of your participation in this study.

You will be reimbursed for reasonable travel costs for the VTE scan visit. Individual researchers will not receive any personal payment over and above normal salary, or any other benefits or incentives, for taking part in this research. The research is being coordinated by Imperial College London, who have overall responsibility for coordination of the study. The research has been reviewed by the National Institute for Health Research, representatives from all of the participating hospitals and organisations, and an independent National Research Ethics Committee, and the Health Research Authority (HRA).

Contact Details

If you have any further questions about your treatment, please discuss them with your doctor. You may also find it helpful to contact the research nurse on XXXXXX.

If you would like further information about clinical research, the UK Clinical Research Collaboration (a partnership of organisations working together on clinical research in the UK) has published a booklet entitled ‘Understanding Clinical Trials’. Contact UKCRC: website: http://www.ukcrc.org/wp-content/uploads/2014/03/iCT_leaflet.pdf

THANK YOU FOR READING THIS INFORMATION SHEET

Imperial College London

CONSENT FORM

GRACE

Site ID:	Initials:
Participant Trial ID:	Principal Investigator Name:

Full Title of Project: GRAduated Compression stocking as an adjunct to Extended duration pharmacological thromboprophylaxis for venous thromboembolism prevention (GRACE Trial).

Study Protocol number: 22HH7932

Is verbal consent being provided via telephone? (please initial one option):

☐ Yes – Please read each statement to the participant. Once the participant agrees to each statement, please provide the initials of the person taking consent (i.e. the researcher) in each box

☐ No – Participant to read the consent form and they write their own initials in each box

Please initial box

1. I confirm that I have read and understand the participant information sheet version .... dated .... for **GRAduated Compression stocking as an adjunct to Extended duration pharmacological thromboprophylaxis for venous thromboembolism prevention (GRACE Trial)** and have had the opportunity to ask questions which have been answered fully.	
2. I understand that my participation is voluntary, and I am free to withdraw at any time, without giving any reason and without my legal rights nor treatment / healthcare being affected.	
3. I understand that sections of any of my medical notes may be looked at by responsible individuals from Imperial College London, from my NHS Trust or hospital site, or from regulatory authorities where it is relevant to my taking part in this research.	

4. I agree to my data (which includes my identifiable data [name, email address and contact telephone numbers]) being entered onto a secure database held at the University of Edinburgh, in accordance with the Data Protection Act 2018 and that members of the research team from Imperial College London will have access to these contact details so that they can contact me.	
5. I understand that if I am eligible for the study, a member of the research team from Imperial College London will have access to my identifiable data to allow contact (via telephone, email, SMS or written) at 7, between 21 and 35 days and 90-days after my surgical procedure to collect follow-up data	
6. I understand that data collected from me are a gift donated to Imperial College and that I will not personally benefit financially if this research leads to an invention and/or the successful development of a new test, medication treatment, product or service.	
7. I understand that my pseudonymised data will be accessed by the University of Edinburgh for the analysis	
8. If during the study my clinical care team determine that I have lost capacity to provide informed consent, I will be withdrawn from the study and any identifiable data collected with consent would be retained and used in the study	
9. I consent to take part in GRACE	

Optional consent section (please initial the appropriate box)

10. I agree to my GP, or any other doctor treating me, being notified of my participation in this study.

Give consent ☐ Do not give consent ☐

11. I agree for information collected about me to be used to support other research or in the development of a new treatment by an academic institution or commercial company in the future, including those outside of the United Kingdom (which Imperial has ensured will keep this information secure).

Give consent ☐ Do not give consent ☐

12. I give/do not give consent for my data to be linked with appropriate national databases, including Hospital Episode Statistics (HES), as well as for longer term follow-up in the event the trial is extended.

Give consent Do not give consent

13. I give/do not give consent to be contacted in the future with regards to this study, should the study be extended.

Give consent ☐ Do not give consent ☐

14. I give / do not give consent to being contacted about the possibility to take part in other research studies.

Give consent ☐ Do not give consent ☐

Name of participant	Signature	Date
Name of person taking consent<br>(if different from Principal Investigator)	Signature	Date

1 copy for participant; 1 copy for Principal Investigator 1 copy for hospital notes

To ensure confidence in the process and minimise risk of loss, all consent forms must be printed, presented and stored in double sided format
