# Supplementary material for: GRACE: protocol for a UK, secondary care, multicentre, assessor-blinded randomised controlled trial with a non-inferiority comparison to evaluate graduated compression stockings as an adjunct to extended duration pharmacological thromboprophylaxis for venous thromboembolism prevention
Source: BMJ Open. 2025 Jul 6;15(7):e095482. doi: 10.1136/bmjopen-2024-095482 (PMC12230952; doi:10.1136/bmjopen-2024-095482)
Supplement: Supplementary file 3 [file bmjopen-2024-095482.reviewer_comments.pdf]

PEER REVIEW HISTORY

BMJ Open publishes all reviews undertaken for accepted manuscripts. Reviewers are asked to complete a checklist review form and are provided with free text boxes to elaborate on their assessment. These free text comments are reproduced below.

ARTICLE DETAILS

Title (Provisional)

GRACE: protocol for a UK, secondary care, multicentre, assessor-blinded randomised controlled trial with a non-inferiority comparison to evaluate graduated compression stockings as an adjunct to extended duration pharmacological thromboprophylaxis for venous thromboembolism prevention

Authors

Lawton, Rebecca; Heatley, Francine; Beggs, Andrew D; Everington, Tamara; Hamady, Zaed; Hunt, Beverley J; Jasionowska, Sara; Kyrgiou, Maria; Liddle, Alexander; Machin, Matthew; Norrie, John; Pinkney, Tom; Rees, J L; Saghdaoui, Layla Bolton; Shalhoub, Joseph; Smith, Sasha; Toh, Simon; Watkin, Nick; Williams, Linda; Davies, Alun

---

VERSION 1 - REVIEW

**Reviewer**	**1**
**Name**	**Cosmi, Benilde**
**Affiliation**	**Angiology and Blood Coagulation Unit, Department of Medical and Surgical Sciences, University of Bologna, Bologna, Italy**
**Date**	**23-Dec-2024**
**COI**	**None**

The authors have planned an Assessor-blinded randomised controlled trial with a non-inferiority comparison to evaluate the potential benefit of Graduated Compression Stockings (GCS) in addition to extended duration pharmacological thromboprophylaxis (EDPTP) for elective surgical patients at highest risk of venous thromboembolism (VTE).

Minor problem: the local policy for duration of GCS (until ambulant, until discharge or post-discharge) should be specified. Infact thromboembolic deterrent stockings (they are firm elastic and not bielastic stockings) are usually worn when patients are bed-ridden and not ambulant. Nowadays, in post-surgical patients early mobilization is encouraged and many patients can recover full mobility very rapidly, thus limiting the usefulness of wearing GCS. The authors should address this issue.

**Reviewer**	**2**
**Name**	**Pizzarossa, Ana Carina**
**Affiliation**	**Hospital de Clínicas Dr Manuel Quintela, Médica C**
**Date**	**09-Mar-2025**
**COI**	**None**

This protocol is of great medical interest and will answer important clinical questions.

After reading it, I have some minor doubts and questions

1 I would like you to include the model consent form and other related documentation given to participants and authorised surrogates

2 Since the duration of use of extended thromboprophylaxis and/or use of compression stockings will be determined by standard clinical practice, I would like to know how you will associate the duration of both treatments to the outcomes.

3 In the references, I would like you to correct the wording of the author in the following citations:

1. Digital N. 5.1 deaths from venous thromboembolism (VTe) related events within 90 days post discharge from hospital. 2019.

21. Collaboration CT. Effectiveness of thigh-length graduated compression stockings to reduce the risk of deep vein thrombosis after stroke (CLOTS trial 1): a multicentre, randomised controlled trial. The Lancet. 2009;373(9679):1958-65.

26 Digital N. Hospital Episode Statistics (HES), Hospital Admitted Patient Care Activity 2022 [cited 2022 03/12/2022]. Available from: https://digital.nhs.uk/data-andinformation/

data-tools-and-services/data-services/hospital-episode-statistics#hespublications.

35 Initiative C. Core Outcome Measures in Effectiveness Trials 2022 [cited 2022 12/16/2022]. Available from: https://www.comet-initiative.org/.

VERSION 1 - AUTHOR RESPONSE

Reviewer 1:

The authors have planned an Assessor-blinded randomised controlled trial with a non-inferiority comparison to evaluate the potential benefit of Graduated Compression Stockings (GCS) in addition to extended duration pharmacological thromboprophylaxis (EDPTP) for elective surgical patients at highest risk of venous thromboembolism (VTE).
Minor problem: the local policy for duration of GCS (until ambulant, until discharge or post-discharge) should be specified. Infact thromboembolic deterrent stockings (they are firm elastic and not bielastic stockings) are usually worn when patients are bed-ridden and not ambulant. Nowadays, in post-surgical patients early mobilization is encouraged and many patients can

recover full mobility very rapidly, thus limiting the usefulness of wearing GCS. The authors should address this issue.

We thank Professor Cosmi for her comments. GRACE is a pragmatic trial and as such the protocol does not specify the length of time that graduated compression stockings should be worn during and after surgery. The study stipulates that each site should follow its own local hospital policy and procedures. These policies also vary depending on the type and complexity of surgery. Sites are asked to record the baseline prescription of graduated compression stockings on the database and patients are asked at follow up if they wore their stockings in line with the advice they were given at baseline. When the results of the study are available, duration of GCS will be reported as part of adherence reporting.

The manuscript has been updated at lines 265-267- *"Depending on the type and complexity of surgery and local hospital policy, duration for the prescription of GCS may be; until ambulant, until discharge, or longer. This will be fully reported in the results publication."*

We also referred to adherence reporting at lines 290-296.

Reviewer 2:

We would like to thank Dr Pizzarossa for taking the time to review this manuscript. We address her comments both here and in the marked version of the text.

1. I would like you to include the model consent form and other related documentation given to participants and authorised surrogates

A copy of the current consent form (version 3.0) was uploaded with the original submission. We have also uploaded a copy of the current Patient Information Sheet (v2.0)

2. Since the duration of use of extended thromboprophylaxis and/or use of compression stockings will be determined by standard clinical practice, I would like to know how you will associate the duration of both treatments to the outcomes.

Depending on the results of the study we will present a descriptive statistical evaluation of adherence to both GCS and thromboprophylaxis.

Further details regarding adherence are found at Lines 290-296.

3 In the references, I would like you to correct the wording of the author in the following citations:

1. Digital N. 5.1 deaths from venous thromboembolism (VTe) related events within 90 days post discharge from hospital. 2019.

There is no individual author for this reference as it is cited from NHS England Digital data. We have amended the manuscript to update the full name as 'NHS Digital' rather than 'Digital N'. We have also updated this reference in the GRACE protocol v2.0.

Please note this is not tracked changed in the marked version of the manuscript as EndNote updates references without tracking.

21. Collaboration CT. Effectiveness of thigh-length graduated compression stockings to reduce the risk of deep vein thrombosis after stroke (CLOTS trial 1): a multicentre, randomised controlled trial. The Lancet. 2009;373(9679):1958-65.

Apologies, reference 21 is found in the GRACE study protocol v2.0 which was included as an appendix. This reference has been updated. We also update the same reference in the manuscript (reference 17) The text in each document has been updated:

Dennis M, Sandercock PA, Reid J, Graham C, Murray G, Venables G, et al. Effectiveness of thigh-length graduated compression stockings to reduce the risk of deep vein thrombosis after stroke (CLOTS trial 1): a multicentre, randomised controlled trial. Lancet. 2009;373(9679):1958-65.

26 Digital N. Hospital Episode Statistics (HES), Hospital Admitted Patient Care Activity 2022 [cited 2022 03/12/2022]. Available from: https://digital.nhs.uk/data-andinformation/ data-tools-and-services/data-services/hospital-episode-statistics#hespublications.

We thank the reviewer for their comment. Reference 26 is from the GRACE study protocol version 2.0. There is no specific author for this reference as it is cited from NHS England Digital data. We have amended to update the full name as 'NHS Digital' rather than 'Digital N'.

This reference also appears as 24 in the manuscript, this has also been updated:

24. NHS Digital. Hospital Episode Statistics (HES), Hospital Admitted Patient Care Activity 2022 [cited 2022 03/12/2022]. Available from: https://digital.nhs.uk/data-and-information/data-tools-and-services/data-services/hospital-episode-statistics#hes-publications

35 Initiative C. Core Outcome Measures in Effectiveness Trials 2022 [cited 2022 12/16/2022]. Available from: https://www.comet-initiative.org/.

Thank you for carefully going through the references. This reference does not appear in our original submitted manuscript; it appears in the GRACE study Protocol v2.0. The authors have updated this reference in the protocol:

35. Williamson PR, Altman DG, Bagley H, Barnes KL, Blazeby JM, Brookes ST, et al. The COMET Handbook: version 1.0. Trials. 2017;18(3):280.
